# Progressive Fatigue-Damage Evolution and Hydraulic Pressure Cycling Life Prediction of Type IV Hydrogen-Storage Cylinders Considering PA6 Liner Viscoelasticity

**DOI:** 10.3390/ma19214577

**Published:** 2026-10-29

**Authors:** Jinjie Lu, Chuanxiang Zheng, Xinfeng Zhang

**Affiliations:** 1 Institute of Process Equipment, College of Energy Engineering, Zhejiang University, Hangzhou 310007, China; 2 School of Information and Electrical Engineering, Hangzhou City University, Hangzhou 310015, China

**Keywords:** Type IV hydrogen-storage cylinder, PA6 liner, viscoelasticity, CFRP, progressive fatigue damage, hydraulic pressure cycling, fatigue life prediction

## Abstract

The hydraulic pressure cycling life of Type IV hydrogen-storage cylinders is jointly governed by the time-dependent response of the liner, local stresses in the overwrap, and progressive damage, and therefore cannot be reliably extrapolated from nominal pressure or specimen-level fatigue life alone. This study addresses two questions: what level of constitutive description must be retained for the polyamide 6 (PA6) liner to obtain a credible local cyclic stress history in the carbon fiber-reinforced polymer (CFRP) overwrap; and whether CFRP specimen-level fatigue data can be transferred to cylinder-level damage and evaluated in terms of life, critical regions, and failure characteristics. Uniaxial tensile and stress–relaxation tests were used to calibrate a PA6 elastoplastic model (P0) and an elastoplastic–viscoelastic model (P1). Based on CFRP static tests and tension–tension fatigue tests at different ply angles, fiber-tension and matrix-dominated fatigue-driving parameters were constructed in the material-coordinate system. A cylinder-level fatigue-damage framework was then established by combining local fatigue-driving-ratio correction, mode-specific S–N relationships, life-fraction accumulation, direction-dependent stiffness degradation, and cycle jumping. With identical cylinder geometry and CFRP parameters, P0 and P1 predicted structural fatigue lives of 17,617 and 21,136 cycles, respectively, corresponding to errors of 22.68% and 7.24% relative to the mean burst life of 22,785 cycles measured for the two burst-tested cylinders. P1 achieved the lower error using 10 material parameters. The simulations showed that matrix-dominated damage developed first; subsequent stiffness degradation shifted fiber-direction load carrying from the inner to the outer plies and ultimately produced a continuous critical region in the dome transition region near the equator. This region corresponded to the circumferential crack-initiation locations observed in the burst-tested cylinders. The proposed framework links the selection of PA6 constitutive fidelity, CFRP specimen-level data, and cylinder-level structural failure, and provides a basis for life assessment and lay-up design of Type IV hydrogen-storage cylinders.

## 1. Introduction

High-pressure gaseous hydrogen storage is essential for extending vehicle range and enabling rapid refueling in fuel-cell vehicles. Type IV hydrogen-storage cylinders, which combine a plastic liner with a fully wrapped carbon fiber-reinforced polymer (CFRP) overwrap, offer low mass, corrosion resistance, and high gravimetric hydrogen-storage density. A typical cylinder comprises a PA6 liner, a CFRP overwrap, and metallic valve bosses. The liner maintains sealing and transmits internal pressure, whereas the CFRP overwrap carries the principal circumferential and axial loads. In the dome, curvature variation, winding-angle variation, and valve-boss constraints create a more complex local stress state than that in the cylindrical section.

Ambient-temperature hydraulic pressure cycling is a core durability test for Type IV cylinders. United Nations Regulation No. 134 (UN R134) specifies cycling between 2 ± 1 MPa and not less than 125% of the nominal working pressure [1]. U.S. Federal Motor Vehicle Safety Standard No. 308 (FMVSS No. 308) likewise specifies a 1–2 MPa to 125–130% nominal-working-pressure range [2]. These protocols define a repeatable mechanical loading boundary for assessing pressure-bearing integrity and fatigue life under cyclic internal pressure [1,2,3]. They do not reproduce all aspects of long-term hydrogen service; rather, they focus on repeated mechanical pressure while limiting the coupled effects of hydrogen permeation, rapid-filling temperature transients, and environmental aging.

Cylinder tests provide the final evidence of hydraulic pressure cycling durability, but their cost, duration, and safety requirements limit the number of design alternatives that can be evaluated experimentally. A development route based only on repeated design, prototype manufacture, pressure cycling, and redesign is therefore inefficient for comparing alternative liner descriptions, overwrap lay-ups, and dome geometries. Material and specimen tests provide the required parameter basis, whereas a cylinder-level finite-element analysis can resolve the evolving local stress state, fatigue damage, and stiffness change that cannot be measured directly within the overwrap. The required engineering capability is consequently a closed analysis-and-test framework: numerical analysis narrows the design space and identifies the critical structural response, while cylinder tests verify fatigue life, critical regions, and post-test failure characteristics.

Existing studies have established important elements of this framework. Lainé et al. combined internal hydraulic loading, full-field displacement measurements, and acoustic-emission monitoring to examine the structural response of composite pressure vessels [4]. Hu et al. analyzed 70 MPa Type IV cylinder failure and validated dome-reinforcement schemes using numerical simulations and burst tests [5,6]. Nebe et al. and Tian et al. showed that stacking sequence and circumferential ply-drop location influence structural response and failure behavior [7,8,9]. Acoustic-emission investigations have further identified internal damage activity during hydraulic loading and burst characteristics of used vessels [10,11], and a recent review summarized available methods for Type IV cylinder failure assessment [12]. These studies clarify how geometry, lay-up, and valve-boss constraints affect static response and burst capacity, but static stress, deformation, and burst pressure do not by themselves determine how damage evolves over tens of thousands of pressure cycles.

At the material level, the PA6 liner is one source of uncertainty in the local cyclic stress history transferred to the CFRP overwrap. Previous studies have investigated the effects of moisture and temperature on PA6 deformation [13,14], viscoelastic modeling under hygrothermal conditions and coupled water-diffusion/stress–relaxation simulations [15,16], and nonlinear viscoelastic–viscoplastic behavior [17]. A rate-independent elastoplastic model can reproduce yielding and hardening, whereas a generalized Maxwell network additionally represents recoverable relaxation during loading, holding, and unloading, at the cost of additional parameters and constitutive integration effort [18]. Material-level curve-fit quality alone, however, does not establish which time-dependent mechanisms must be retained to provide a credible cylinder-level fatigue prediction.

At the overwrap level, Hashin and Hashin-Rotem proposed combined-stress formulations that distinguish fiber- and matrix-related modes [19,20,21], while Puck and Schürmann developed a physically based failure analysis method for composite laminates [22]. Lin et al. and Jin et al. applied progressive failure analysis to static load-bearing and burst studies of composite pressure vessels [23,24]. Off-axis fatigue studies further demonstrated that ply angle, stress ratio, and the dominant load-carrying mode jointly affect composite fatigue life [25,26]. Progressive fatigue models, local fatigue-driving-ratio corrections, and cycle-jump strategies provide routes for combining life-fraction accumulation, stiffness degradation, and computational efficiency [27,28,29,30]. For a filament-wound cylinder, however, ply angle, through-thickness position, dome contour, and valve-boss constraints cause local cyclic stress states to vary between material points; stiffness degradation then changes those states in subsequent cycles.

Despite this foundation, three interdependent gaps remain for hydraulic pressure cycling life prediction of actual Type IV cylinders. First, PA6 constitutive models are generally selected from material-level response, but the constitutive fidelity required to obtain a credible CFRP local cyclic stress history and cylinder life at an acceptable computational cost remains insufficiently defined. Second, static failure criteria, S–N relationships, and stiffness-degradation models are available, but they require a consistent material-coordinate interface to transfer specimen-level fatigue data to the evolving local state of a filament-wound structure. A prescribed nominal pressure ratio or initial peak-valley stress field cannot replace the local fatigue-driving ratio and stiffness feedback at each integration point. Third, cylinder-level validation requires more than agreement in life alone. A predictive framework should also identify the critical region and provide a mechanically consistent interpretation of the observed failure characteristics. These three gaps are sequential: liner fidelity affects the local stress history, the local stress history drives fatigue damage and stiffness loss, and the resulting structural response must be assessed against cylinder-test evidence.

Accordingly, this study establishes a linked PA6–CFRP–cylinder analysis framework for hydraulic pressure cycling. First, PA6 elastoplastic (P0) and elastoplastic–viscoelastic (P1) models are calibrated from tensile and stress–relaxation tests under a moisture condition representative of the actual liner; their cylinder-level effects are then compared using identical geometry, pressure history, and CFRP parameters. Second, static and tension–tension fatigue tests of 0° and off-axis CFRP specimens are used to construct fiber-tension and matrix-dominated fatigue-driving parameters, mode-specific S-N relationships, local fatigue-driving-ratio correction, life-fraction accumulation, direction-dependent stiffness degradation, and cycle jumping. Third, the resulting cylinder-level simulations are evaluated against hydraulic pressure cycling life, the final critical region, and post-test failure morphology of actual V-1 cylinders. This technical route links material parameters to structural fatigue response, reduces reliance on trial-and-error prototype testing, and provides an analysis basis for Type IV cylinder life assessment and lay-up design.

## 2. Materials and Methods

### 2.1. PA6 Liner Material Condition and Testing

The moisture condition of the PA6 liner directly affects the constitutive parameters identified from mechanical tests. PA6 is a hygroscopic semicrystalline polyamide. Water absorbed in its amorphous phase weakens intermolecular interactions and increases segmental mobility [31,32]; consequently, the elastic modulus, yield strength, post-yield flow stress, and cyclic response vary with water uptake [13,14,33,34,35]. The material condition used here was therefore defined by a water uptake representative of the actual liner, providing a unified material-state basis for identifying the elastoplastic and viscoelastic parameters used in the subsequent cylinder-level analysis.

**Figure 1:**
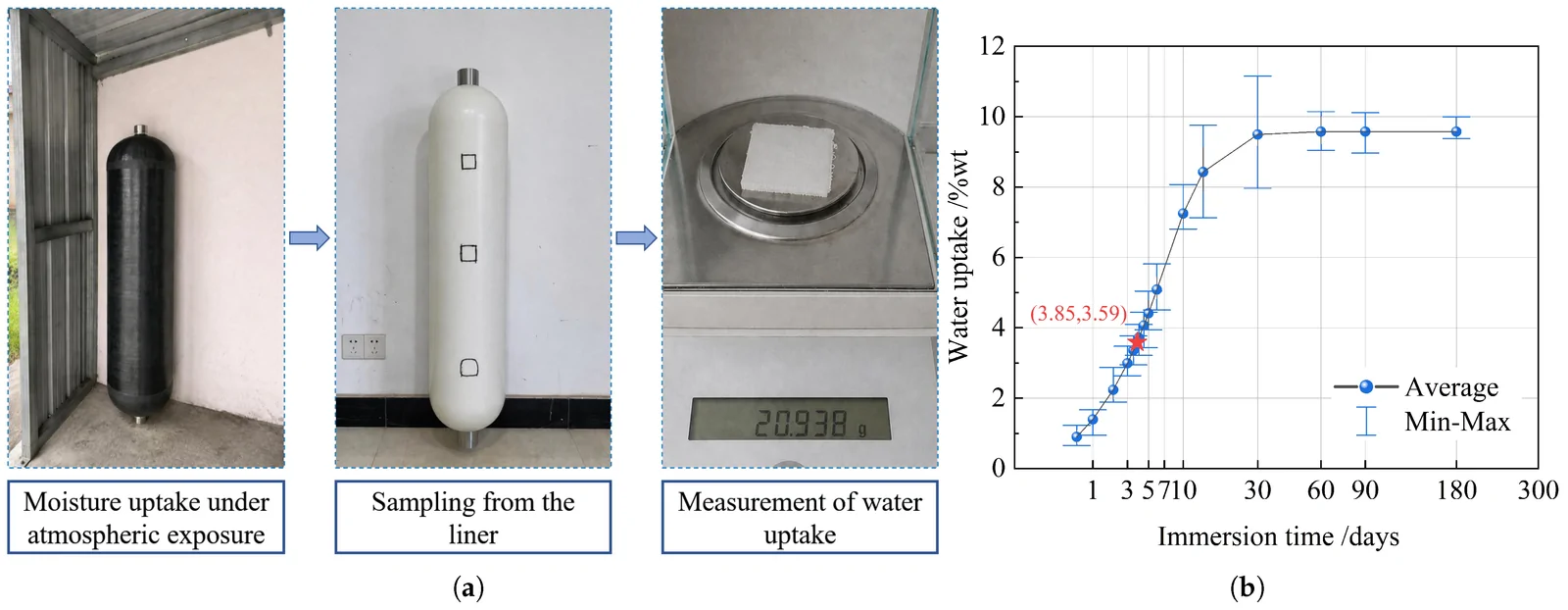
Moisture condition of PA6 and specimen conditioning process: (**a**) measurement of the moisture condition of the actual cylinder liner; (**b**) water-bath conditioning history of PA6 specimens.

Liner samples with approximate dimensions of 50mm×50mm×7mm were taken from an actual PA6 cylinder liner that had been stored in a sheltered outdoor environment and exposed to the atmosphere for approximately one year. According to the water-absorption test standard [36], the measured mass water uptake of the actual liner was 3.59 ± 0.16 wt.%. As shown in Figure 1a, samples were taken directly from the long-term atmosphere-exposed liner and weighed to establish the conditioning target. Test specimens were prepared from the same Akulon® F223-D PA6 pellets used for the liner and conditioned in a room-temperature water bath. As shown in Figure 1b, water uptake increased rapidly at first and then gradually leveled off. At approximately 3.85 d, the specimen water uptake matched the 3.59 ± 0.16 wt.% value measured for the actual liner; this duration was therefore selected for the subsequent mechanical tests. The water bath was used to match the measured total water uptake rather than to reproduce the complete outdoor environmental history of the liner. All subsequent tests were conducted at 23 °C and 50% relative humidity, with specimen conditioning and environmental control following the relevant standard [37].

Uniaxial tensile tests were used to identify the initial stiffness, 0.2% offset yield strength, post-yield hardening, and Poisson’s ratio of PA6, with the test conditions defined according to the relevant tensile-testing standard [38]. Engineering stress-engineering strain data were recorded and converted to true stress and logarithmic strain for finite-element constitutive integration. Stress–relaxation tests were performed under a small-strain hold condition: specimens were rapidly loaded to 0.1% axial strain and held for 6 h (21,600 s), while the load decay was continuously recorded. The relaxation modulus was calculated from the load, initial cross-sectional area, and prescribed hold strain. To ensure comparability between P0 and P1, both models were calibrated using data obtained under the same material condition.

### 2.2. CFRP Static and Tension–Tension Fatigue Tests

The CFRP overwrap material was an SYT49S-grade carbon-fiber/epoxy system. Selected orthotropic elastic and static-strength parameters were obtained from 0° and 90° tensile tests, V-notched beam shear tests, and Naval Ordnance Laboratory (NOL) ring tests. The parameters not measured directly in the present study, namely $G_{23}$, $\nu_{23}$, $X_{c}$, $Y_{t}$, and $Y_{c}$, were supplemented using lamina properties reported by Wei et al. [39]. The 0° and ±θ off-axis specimens were used to construct the tension–tension fatigue dataset. The static, shear, ring, and tension–tension fatigue tests were conducted in accordance with the corresponding test standards [40,41,42,43,44]. The principal specimen information is summarized in Table 1. The tension–tension fatigue tests were force-controlled at a specimen reference stress ratio of $R_{0}=0.1$. The 5 and 15 Hz loading frequencies were selected in accordance with the loading-frequency provisions of GB/T 35465.1-2017 and GB/T 35465.3-2017 [41,42]. The higher stress levels were tested at 5 Hz. For lower stress levels, which produced longer fatigue lives, the frequency increased to 15 Hz to control the total test duration. During the tests, a forced ambient-air stream was continuously directed across the specimen surface to limit loading-induced temperature rise and minimize temperature-related coupling effects. Differences among the 0°, ±15°, ±30°, and ±45° specimens were used to distinguish the relative contributions of fiber tension, transverse matrix response, and in-plane shear.

### 2.3. Hydraulic Pressure Cycling Tests of the Type IV Cylinder

**Figure 2:**
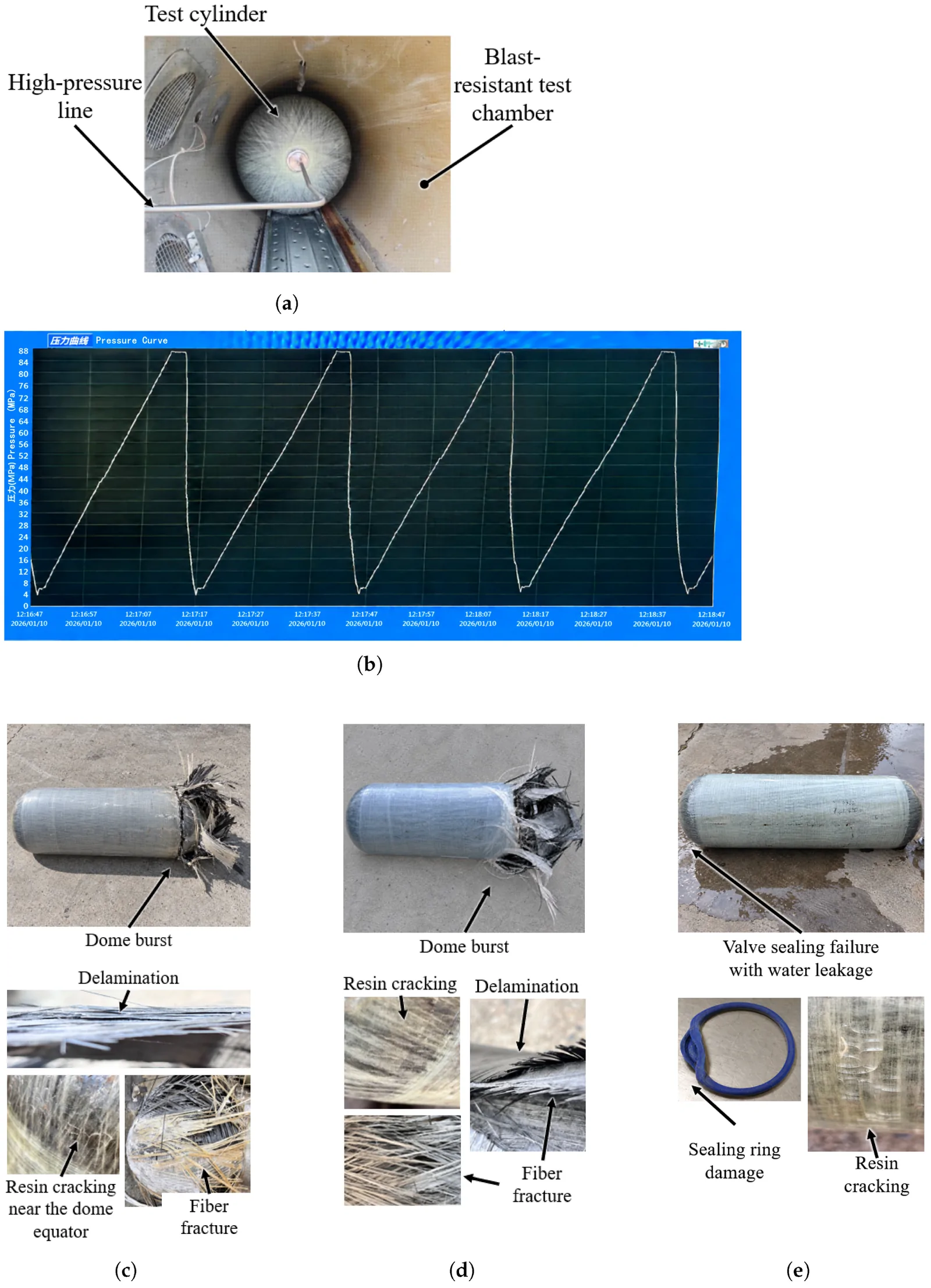
Hydraulic pressure cycling tests and post-test appearance of the V-1 cylinders: (**a**) hydraulic pressure cycling apparatus and test cylinder; (**b**) pressure-time history; (**c**) overall and local burst-failure morphology of cylinder #1; (**d**) overall and local burst-failure morphology of cylinder #2; (**e**) overall and local leakage-failure morphology of cylinder #3.

The test cylinder, designated V-1, comprised a PA6 liner, a fully wrapped CFRP overwrap, and two Al6061-T6 valve bosses. Hydraulic pressure cycling was conducted between 2 and 87.5 MPa. The upper pressure of 87.5 MPa is 125% of the 70 MPa nominal working pressure, consistent with the ambient-temperature hydraulic pressure cycling upper limit specified in UN R134 [1] and within the 125–130% nominal-working-pressure range specified in FMVSS No. 308 [2]. The test equipment, pressure-time history, and post-test appearance are shown in Figure 2, and the complete test records for the three cylinders are listed in Table 2.

As shown in Figure 2a, each test cylinder was placed in a blast-resistant test chamber and hydraulically loaded through a high-pressure line to isolate the burst hazard and maintain a controlled pressure boundary. The repeated peak-valley pressure history in Figure 2b subjected all cylinders to the same 2–87.5 MPa cyclic pressure range. Both burst-tested cylinders in Figure 2c,d exhibited circumferential cracking in the dome transition region near the equator, accompanied by resin cracking, fiber-bundle fracture, and local delamination. These observations indicate that the geometry, ply angles, and local load transfer in this transition region form a persistent structural weak location and that final failure involves multiple mechanisms associated with the matrix, interfaces, and fiber load carrying. In contrast, cylinder #3 in Figure 2e first leaked at the valve seal, where sealing-ring damage was also observed. Its CFRP load-bearing overwrap did not reach the same overall burst termination state as the cylinders in Figure 2c,d, indicating a competing termination mode distinct from CFRP structural burst.

On this basis, Section 6 evaluates CFRP structural life, critical regions, and failure morphology using only the two burst-tested cylinders shown in Figure 2c,d. The result for Figure 2e is retained as evidence of a valve-seal failure mode that is outside the scope of the present model.

The first two cylinders terminated by burst of the CFRP load-bearing structure, giving a measured mean burst life of (23,213 + 22,357)/2 = 22,785 cycles. The third cylinder leaked at the valve after 20,005 cycles and therefore does not represent the structural failure endpoint defined by the present intralaminar progressive fatigue model for the CFRP. Because the model does not include seal-contact failure or leakage criteria at the valve, the result for the third cylinder is retained as evidence defining the applicability of the prediction framework but is not combined with the burst-life predictions when calculating the mean.

## 3. Constitutive Models and Parameter Calibration for the PA6 Liner

### 3.1. Elastoplastic Baseline Model (P0)

P0 uses isotropic linear elasticity, a $J_{2}$ (von Mises) yield criterion, an associated flow rule, and linear isotropic hardening. It serves as the baseline model without independent time-dependent internal variables and describes the elastic response, onset of yielding, and average post-yield hardening trend of PA6 during the initial monotonic loading stage. P1 extends this formulation with a viscoelastic network to represent stress relaxation during strain holding. Parameters are defined in the true stress–logarithmic strain space to remain consistent with the material integration used in Abaqus2020/Standard. The yield function is given in Equation (1).

(1) $$f\left(\sigma ,{\bar{\varepsilon }}^{p}\right)=\sigma_{\mathrm{eq}}-\left[\sigma_{y0}+H{\bar{\varepsilon }}^{p}\right]⩽0,\sigma_{y0}=29.76\;\mathrm{MPa},\;H=41.41\;\mathrm{MPa}$$

where, *f* is the yield function of the Cauchy stress tensor σ, $\sigma_{\mathrm{eq}}$ is the von Mises equivalent stress, $\sigma_{y0}$ is the initial yield strength, *H* is the linear isotropic hardening modulus, and ${\bar{\varepsilon }}^{p}$ is the accumulated equivalent plastic strain. In the present P0/P1 calibration, $\sigma_{y0}$ is equal to the 0.2% offset yield strength $\sigma_{y}=29.76$ MPa reported in Table 3.

**Figure 3:**
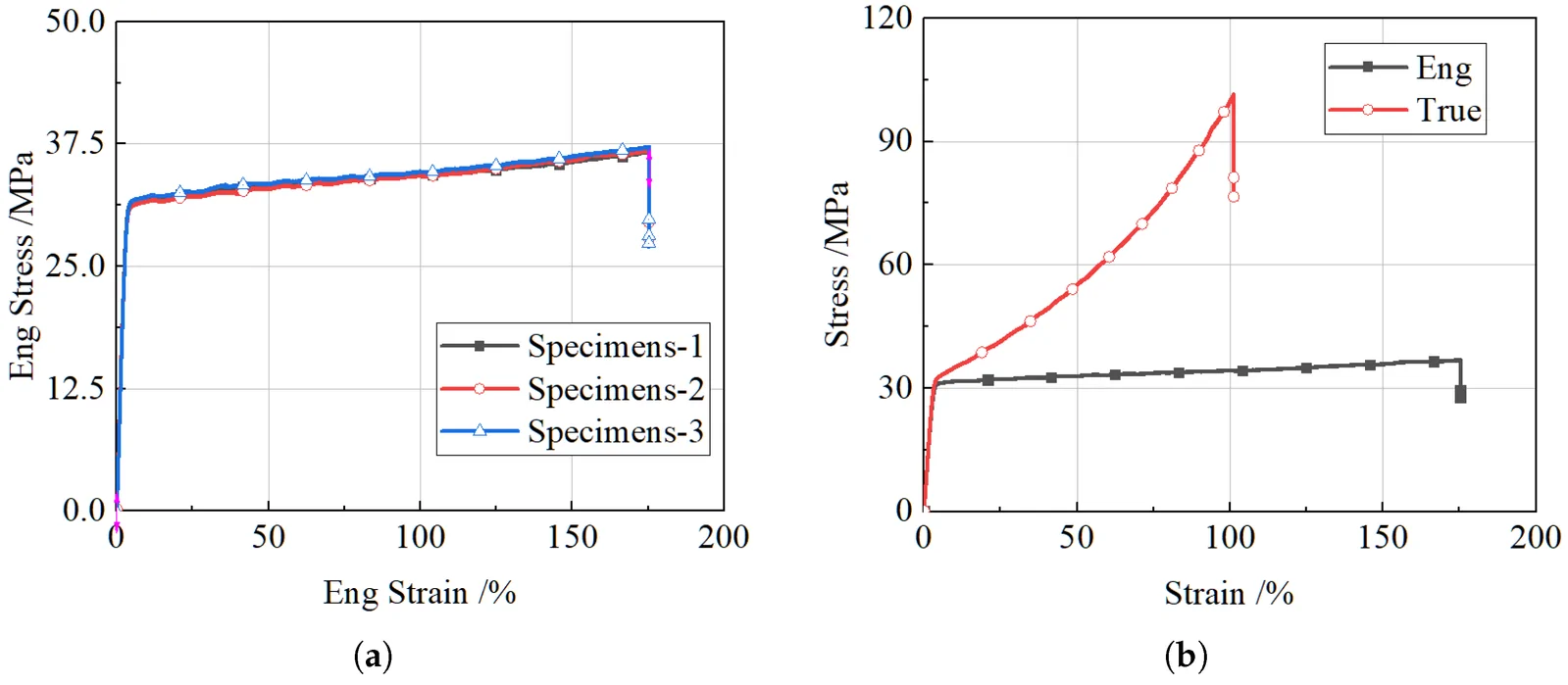
PA6 tensile response and conversion between engineering and true stress–strain measures: (**a**) uniaxial engineering stress–strain response; (**b**) conversion from engineering stress–strain to true stress–logarithmic strain.

The initial elastic stiffness is defined by E=978.21 MPa and ν=0.35. Once the yield surface is reached, the plastic strain and stress are updated using an implicit return-mapping scheme. The monotonic tensile response and the relationship between engineering and true stress–strain measures are shown in Figure 3, and the baseline calibration values are listed in Table 3. The constitutive implementation was performed through an Abaqus2020/Standard user material subroutine (UMAT).

As shown in Figure 3a, the three engineering stress–strain curves almost coincide in terms of initial stiffness, yield point, and post-yield evolution, indicating good repeatability of the monotonic tensile response of PA6 at the current moisture condition. The load continues to increase gradually after yielding; accordingly, P0 uses linear isotropic hardening to represent the average hardening trend. As tensile deformation increases, the current cross-sectional area decreases relative to the initial area, causing the difference between true and engineering stress in Figure 3b to increase progressively and resulting in a substantially steeper hardening slope when identified in true-stress space. The UMAT therefore uses true stress–logarithmic strain parameters so that the stress measure used in material calibration remains consistent with that used in finite-strain constitutive integration.

### 3.2. Coupled Elastoplastic–Viscoelastic Model (P1)

To represent the recoverable time-dependent response of PA6 during pressure holding and unloading, P1 places a three-branch generalized Maxwell nonequilibrium network in parallel with the elastoplastic part of P0. The equilibrium network updates yield, hardening, and irreversible plastic strain, whereas the Maxwell network is driven by the recoverable deviatoric strain increment after subtraction of the plastic strain increment and represents viscoelastic stress relaxation over different characteristic times. The viscoelastic mechanism is applied only to the deviatoric response, while the volumetric response remains elastic. Previous work on the viscoelastic response of PA6, identification of generalized Maxwell networks, and deviatoric viscoelastic formulations provides the basis for this modeling choice [15,16,18,45,46].

In this study, a material point refers to an element integration point at which the UMAT is called. Local stresses, consumed life fractions, stiffness-degradation variables, and internal variables are stored and updated at the integration points. The relaxation modulus, total-stress decomposition, and exponential integration of the branch deviatoric stresses in the generalized Maxwell network are given in Equations (2)–(4), respectively.

(2) $$E\left(t\right)=E_{\infty }+\sum_{i=1}^{3}E_{i}\exp\left(-\frac{t}{\tau_{i}}\right).$$

where, E(t) is the relaxation modulus at elapsed time *t*; $E_{\infty }$, $E_{i}$, and $\tau_{i}$ are the long-term modulus, modulus, and characteristic relaxation time of the *i*th Maxwell branch, respectively. The measured relaxation-modulus history was fitted to Equation (2) using the parameter vector $\vartheta =[E_{\infty },E_{1},E_{2},E_{3},\tau_{1},\tau_{2},\tau_{3}]$. A bounded nonlinear least-squares procedure with equal point weights was used. The final feasible solution was selected by minimizing the normalized residual $J_{\mathrm{rel}}=\sum_{k}{\left[\left(E\left(t_{k};\vartheta \right)-{\bar{E}}_{\exp}\left(t_{k}\right)\right)/{\bar{E}}_{\exp}\left(t_{k}\right)\right]}^{2}$, where $t_{k}$ is the *k*th experimental time point, $E(t_{k};\vartheta )$ is the fitted relaxation modulus, and ${\bar{E}}_{\exp}\left(t_{k}\right)$ is the mean measured relaxation modulus at that time. The constraints were $0\leq E_{\infty }\leq 2000$ MPa, $0\leq E_{i}\leq 1500$ MPa, $0.01\leq \tau_{1}\leq 100$ s, $0.1\leq \tau_{2}\leq 5000$ s, and $1\leq \tau_{3}\leq 100,000$ s. The fitted branches were reported in ascending order of $\tau_{i}$ to avoid ambiguity in branch labeling.

(3) $$\sigma_{n+1}=\sigma_{\mathrm{equilibrium},n+1}+\sum_{i=1}^{3}q_{i,n+1}.$$

(4) $$q_{i,n+1}=a_{i}q_{i,n}+2G_{i}c_{i}\Delta e_{\mathrm{dev}},\;a_{i}=\exp\left(-\frac{\Delta t}{\tau_{i}}\right),\;c_{i}=\frac{1-a_{i}}{\Delta t/\tau_{i}}.$$

where, $\sigma_{n+1}$ is the total Cauchy stress tensor at the end of the increment, $\sigma_{\mathrm{equilibrium}}$ is the stress tensor of the equilibrium network, and $q_{i}$ is the deviatoric stress tensor of the *i*th Maxwell branch. $G_{i}=E_{i}/\left[2\left(1+\nu \right)\right]$. The subscripts *n* and n+1 denote the beginning and end of the current material-integration increment. In Equation (4), Δt is the physical duration of that increment, and $a_{i}$ and $c_{i}$ are the exponential-integration coefficients of the *i*th Maxwell branch. $\Delta e_{\mathrm{dev}}$ is obtained from the recoverable strain increment in the current step by first subtracting the irreversible plastic strain increment and then taking the deviatoric part.

During a hold step, when $\Delta e_{\mathrm{dev}}=0$, $q_{i}$ decays exponentially with time. Equation (4) uses exponential integration under the assumption of a constant strain rate within the time increment to update the history variables of each Maxwell branch.

**Figure 4:**
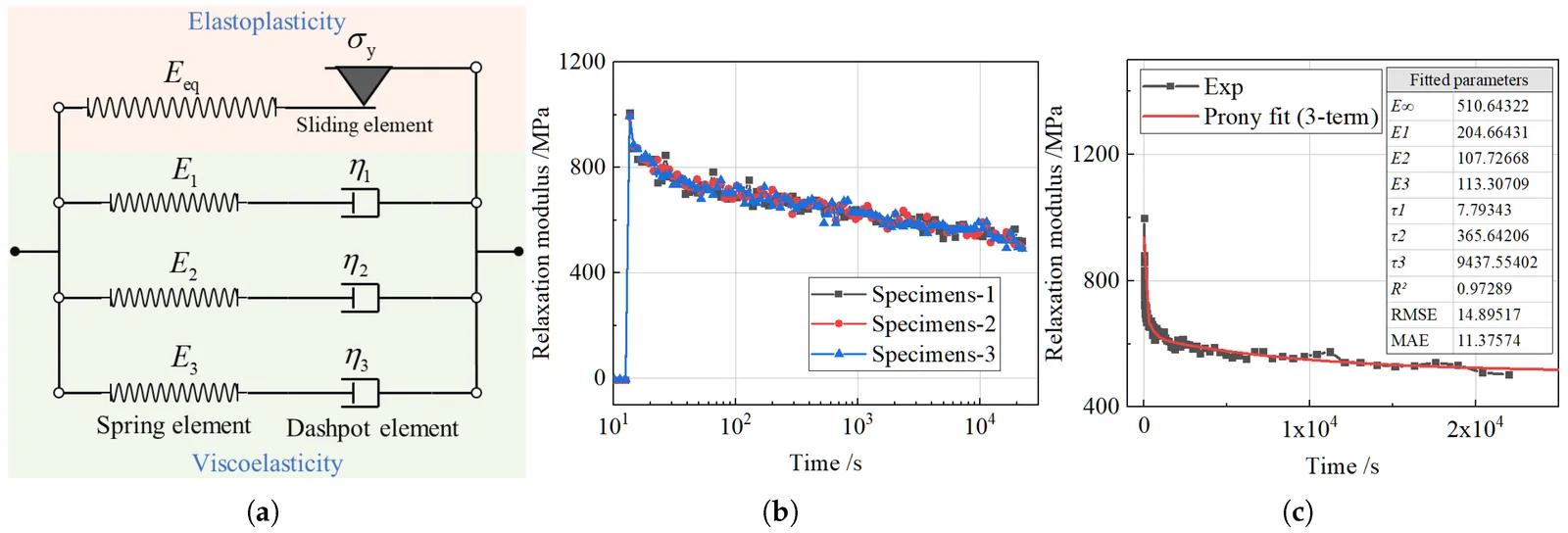
Coupled elastoplastic–viscoelastic P1 representation and identification of PA6 relaxation parameters over the 6 h relaxation window: (**a**) P1 consisting of an elastoplastic equilibrium network and three viscoelastic Maxwell branches; (**b**) PA6 relaxation-modulus tests; (**c**) Prony-parameter identification results.

The three-branch parameters identified from the stress–relaxation tests are listed in Table 4. To retain the initial total stiffness obtained from monotonic tensile calibration, the equilibrium-network modulus used in the three-dimensional P1 implementation is $E_{\mathrm{eq}}=E-\sum_{i}E_{i}=552.51$ MPa. Thus, $E_{\infty }=510.64$ MPa is the long-term modulus of the one-dimensional relaxation fit, whereas $E_{\mathrm{eq}}$ is the equilibrium-network modulus used in the three-dimensional implementation. The former represents the fitted long-time response of Equation (2); the latter maintains the calibrated initial total stiffness after the branch contributions are assembled. The two quantities have different definitions and are not interchangeable. The fit-quality indicators in Table 4 are the coefficient of determination $R^{2}$, root-mean-square error (RMSE), and mean absolute error (MAE). The coupled P1 representation, relaxation data, and fitted curves are shown in Figure 4.

Figure 4a presents the coupled P1 architecture. Its elastoplastic equilibrium network represents the P0-type elastoplastic response, whereas the three Maxwell branches provide the recoverable relaxation component over short-, intermediate-, and long-time intervals. Figure 4b presents the three measured 6 h relaxation-modulus histories. All three specimens exhibit a rapid initial decrease followed by a slower long-time decay, providing a consistent experimental basis for parameter identification. Figure 4c compares the three-branch Prony fit with the measured relaxation-modulus history. The fitted curve reproduces both the initial decay and the subsequent gradual relaxation, with $R^{2}=0.97289$, RMSE =14.89517 MPa, and MAE =11.37574 MPa. The fitted values $\tau_{1}=7.79$ s, $\tau_{2}=365.64$ s, and $\tau_{3}=9437.55$ s provide separated short-, intermediate-, and long-time characteristic scales within the 6 h experimental window. Compared with a single-branch representation, the three-branch form covers a broader relaxation-time window. Compared with higher-order representations, it retains fewer parameters and better parameter identifiability. Increasing the number of Maxwell branches can reduce the fitting residual, but when relaxation data are limited, it can also reduce parameter identifiability and increase the risk of overfitting [18,47,48]. Three branches were consequently adopted to represent the observed relaxation-time spectrum while retaining a parameter set suitable for the subsequent three-dimensional finite-element implementation.

## 4. Mode-Specific Progressive Fatigue-Damage Model for CFRP

**Figure 5:**
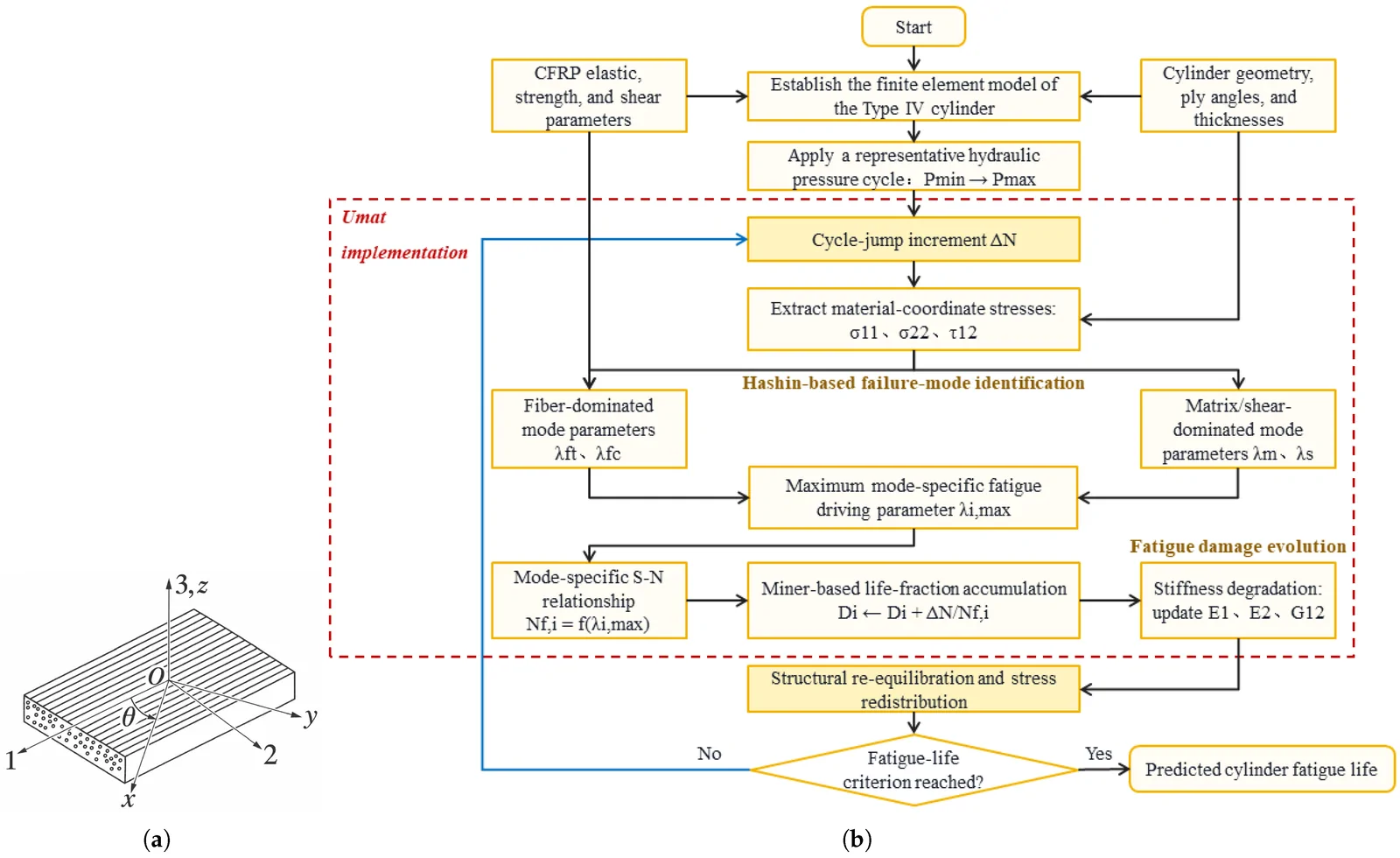
CFRP material-coordinate system and mode-specific fatigue calculation procedure: (**a**) material-coordinate system of the overwrap [49]; (**b**) mode-specific fatigue calculation procedure. Orange-bordered boxes denote model inputs, computational operations, and outputs; blue feedback arrows denote the return of the updated local state to the next cycle-jump increment; the red dashed box delineates the UMAT implementation; and black arrows indicate the sequential calculation flow.

This section establishes the computational procedure for transferring specimen-level cyclic data to damage updates at integration points in the filament-wound structure. In the material-coordinate system shown in Figure 5a, directions 1, 2, and 3 are fixed to the fiber, in-plane transverse, and through-thickness directions, respectively. This allows specimens with different ply angles and the actual overwrap to be compared using the same set of local stress components and prevents differences in the external loading coordinates from obscuring the fiber- and matrix-dominated mechanisms. The computational chain shown in Figure 5b further closes mode-specific S-N life, life-fraction accumulation, and direction-dependent stiffness degradation into finite-element re-equilibration because a reduction in local stiffness changes the stress distribution in the next cycle block; the entire life therefore cannot be accumulated from the initial stress field in a single step. Cycle jumping is used only to accelerate the progression of repeated pressure cycles and does not alter this damage-stiffness-stress feedback relationship.

### 4.1. Mode-Specific Fatigue Representation in the Material-Coordinate System

For an ideal lamina specimen whose fiber direction forms an angle θ with the uniaxial loading direction, the applied axial stress $\sigma_{x}$ is resolved into the material-coordinate system according to Equation (5):

(5) $$\sigma_{11}=\sigma_{x}\cos^{2}\theta ,\;\sigma_{22}=\sigma_{x}\sin^{2}\theta ,\;\tau_{12}=-\sigma_{x}\sin\theta \cos\theta .$$

where, $\sigma_{x}$ is the applied axial stress, θ is the ply angle between the fiber direction and the loading direction, and $\sigma_{11}$, $\sigma_{22}$, and $\tau_{12}$ are the longitudinal, transverse, and in-plane shear stress components in the material-coordinate system, respectively.

Equation (5) illustrates the variation of local stress components with ply angle. For the 0°, ±15°, ±30°, and ±45° laminate specimens, stresses and strains in the material-coordinate system of each ply were obtained from the measured axial load-strain response using classical laminate theory (CLT) and the actual specimen lay-up. These quantities were then used to calibrate the mode-specific S-N relationships and stiffness-degradation parameters. For the Type IV cylinder, the finite-element equilibrium solution provides the stress state at each integration point; the UMAT then transforms this state into the material-coordinate system according to the local material orientation and invokes the calibrated fatigue parameters to update the consumed life fraction and stiffness. The former route identifies parameters from specimen tests, while the latter tests their transfer to life prediction in the wound structure. The two routes are connected by the same definitions of local stress and failure-mode identification [49,50,51].

A three-dimensional orthotropic linear elastic relation is adopted. The compliance matrix $S_{0}$ is constructed from the engineering constants and satisfies the Poisson-ratio reciprocity condition $\nu_{ij}/E_{i}=\nu_{ji}/E_{j}$. The stiffness matrix and stress-increment update for an undamaged material point are given by Equation (6):

(6) $$C_{0}=S_{0}^{-1},\;\Delta \sigma =C_{0}:\Delta \varepsilon .$$

where $S_{0}$ and $C_{0}$ are the undamaged compliance and stiffness matrices, respectively, and Δσ and Δε are the stress and strain increments in the current material-integration step.

The three-dimensional engineering constants and intralaminar static strengths are listed in Table 5. The same set of undamaged material parameters is used for the subsequent strength normalization and fatigue-damage calculations. $E_{1}$, $E_{2}$, $E_{3}$, $G_{12}$, $G_{13}$, $\nu_{12}$, $\nu_{13}$, $X_{t}$, and $S_{12}$ were obtained from the corresponding tests in this study, whereas $G_{23}$, $\nu_{23}$, $X_{c}$, $Y_{t}$, and $Y_{c}$ were taken from the published parameters in Ref. [39]. Both specimen-level calibration and cylinder-level analysis use the same SYT49S-grade carbon-fiber/epoxy material system.

The strength parameters $X_{t}$, $X_{c}$, $Y_{t}$, $Y_{c}$, and $S_{12}$ denote, respectively, tensile and compressive strength in the fiber direction, tensile and compressive strength in the in-plane transverse direction, and in-plane shear strength. They are used to normalize the stresses in the mode-specific formulation; $\lambda_{i}=1$ is not used directly as a cycle-to-failure criterion for a material point.

Different failure modes are governed by different local stress components and corresponding static strengths. The Hashin and Hashin-Rotem criteria use combined stresses to distinguish fiber- and matrix-related failure modes [19,20,21]. To place fiber tension, fiber compression, transverse matrix response, and in-plane shear on a common dimensionless basis, the strength-utilization concept in Ref. [52] is adopted. A homogeneous quadratic failure function $F_{i}$ is converted into a normalized quantity that varies linearly with proportional loading through $\lambda_{i}=\sqrt{F_{i}}$. For the intralaminar stresses that are most relevant under positive internal-pressure cycling, the mode-specific normalized parameters are defined by Equation (7):

(7) $$\begin{array}{cccc} \lambda_{ft} & =\sqrt{{\left(\frac{\sigma_{11}}{X_{t}}\right)}^{2}+\alpha {\left(\frac{\tau_{12}}{S_{12}}\right)}^{2}}, & \; & \sigma_{11}⩾0,\;\alpha =1;\\ \lambda_{fc} & =\frac{|\sigma_{11}|}{X_{c}}, & \; & \sigma_{11}<0;\\ \lambda_{m} & =\sqrt{{\left(\frac{\langle \sigma_{22}\rangle }{Y_{t}}\right)}^{2}+{\left(\frac{\langle -\sigma_{22}\rangle }{Y_{c}}\right)}^{2}+{\left(\frac{\tau_{12}}{S_{12}}\right)}^{2}}; & & \\ \lambda_{s} & =\frac{|\tau_{12}|}{S_{12}}, & \; & \langle x\rangle =\max(x,0). \end{array}$$

where, ft, fc, *m*, and *s* denote the fiber-tension, fiber-compression, matrix-dominated, and independent in-plane shear modes, respectively.

The fiber-tension and matrix-dominated indicators both include the relevant contribution of in-plane shear. $\lambda_{s}$ is retained as an independent pure-shear diagnostic output; therefore, the shear term embedded in $\lambda_{m}$ must not be interpreted as a separately calibrated shear fatigue-life curve. Because the hydraulic pressure cycling considered here is dominated by tensile response under positive internal pressure, fatigue-life progression is restricted to the ft and *m* modes, for which independent fatigue-test calibration is available. $\lambda_{fc}$ and $\lambda_{s}$ are retained for peak-strength diagnostics and future model extension and do not participate in the present life-fraction accumulation, stiffness degradation, or structural endpoint criterion.

To compare the relative contributions of the modes at different ply angles, the relative driving coefficient in Equation (8) is further introduced:

(8) $$\rho_{i}=\frac{\lambda_{\max,i}}{\max_{j}\lambda_{\max,j}}.$$

where $\lambda_{\max,i}$ is the peak fatigue-driving parameter of mode *i* under a given loading condition, and *j* enumerates the evaluated failure modes at the same material point.

**Figure 6:**
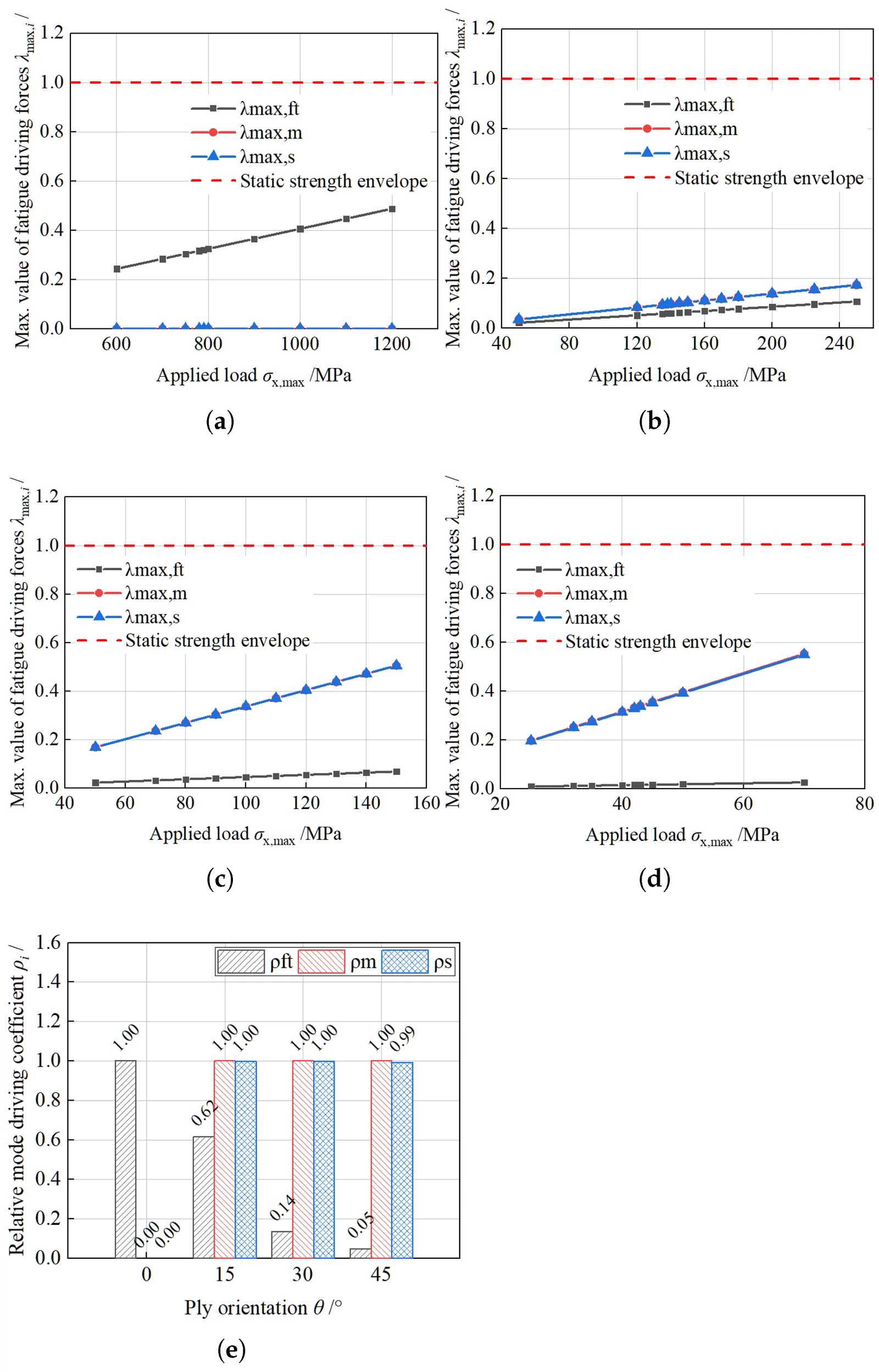
Mode-specific fatigue representation of CFRP specimens with different ply angles: (**a**) peak fatigue-driving parameters for the 0° specimen; (**b**) peak fatigue-driving parameters for the ±15° specimen; (**c**) peak fatigue-driving parameters for the ±30° specimen; (d) peak fatigue-driving parameters for the ±45° specimen; (**e**) relative mode-driving coefficients.

$\rho_{i}$ is used only to identify the relative peak level of each mode under the same loading condition. Previous off-axis fatigue studies have shown that ply angle, local stress ratio, and the dominant load-carrying mode jointly affect laminate fatigue life [25,26,50,51]. As shown in Figure 6a, the load in the 0° specimen is transmitted almost entirely along the fiber direction, and $\lambda_{ft}$ is substantially larger than the matrix and shear indicators; fiber tension is therefore the dominant mode. Once the fiber direction deviates from the applied load, the axial load is resolved simultaneously into longitudinal, transverse, and shear components. Consequently, the ±15° specimen in Figure 6b exhibits comparable relative contributions from ft, *m*, and *s*, indicating competition among multiple mechanisms. As the off-axis angle increases further, the fiber-direction component decreases while the transverse-shear contribution increases, causing the ±30° and ±45° specimens in Figure 6c,d to become matrix-dominated. The relative driving coefficients in Figure 6e quantify this transition: $\rho_{ft}$ decreases from 1 at 0° to approximately 0.62, 0.14, and 0.05 at ±15°, ±30°, and ±45°, respectively, while the m/s contributions approach the controlling level in the off-axis specimens. Based on this physical separation, the 0° data are used to calibrate the ft mode, the ±30° and ±45° data are used to calibrate the *m* mode, and the ±15° data are retained as a mixed-mechanism check rather than being forced into a single dominant S-N fit.

### 4.2. Mode-Specific Life Relationships, Stiffness Degradation, and Cycle Jumping

Each S-N relationship uses only valid finite-life data for its corresponding dominant mode. Before fitting, records involving grip failure, slippage, failure outside the gauge section, or results clearly inconsistent with the trend at the same stress level were removed; this included the anomalous 0° record at $\sigma_{x,\max}=600$ MPa and $N_{f}=65$ cycles. Runouts were retained as right-censored observations in the life plots but were not entered into the ordinary least-squares fit as known failure lives. A Grubbs test (α=0.05) identified no additional statistical outliers requiring removal. The final 0° dataset contained 47 valid fitting points, while the ±30° and ±45° matrix-dominated datasets contained 60 points in total. The mode-specific life equation is given by Equation (9), with the fitted parameters listed in Table 6:

(9) $$\log_{10}N_{f,i}=A_{i}-B_{i}\log_{10}\left(\lambda_{eq,i}\right),\;i\in \Omega =\left\{ft,m\right\}.$$

where $N_{f,i}$ is the predicted fatigue life of mode *i*, $A_{i}$ and $B_{i}$ are the fitted intercept and slope of the corresponding S–N relationship, and Ω={ft,m} is the set of actively calibrated modes. $\lambda_{eq,i}$ denotes the equivalent fatigue-driving parameter mapped to the specimen reference stress ratio $R_{0}=0.1$; its construction from the local cyclic state is given subsequently in Equation (11).

**Figure 7:**
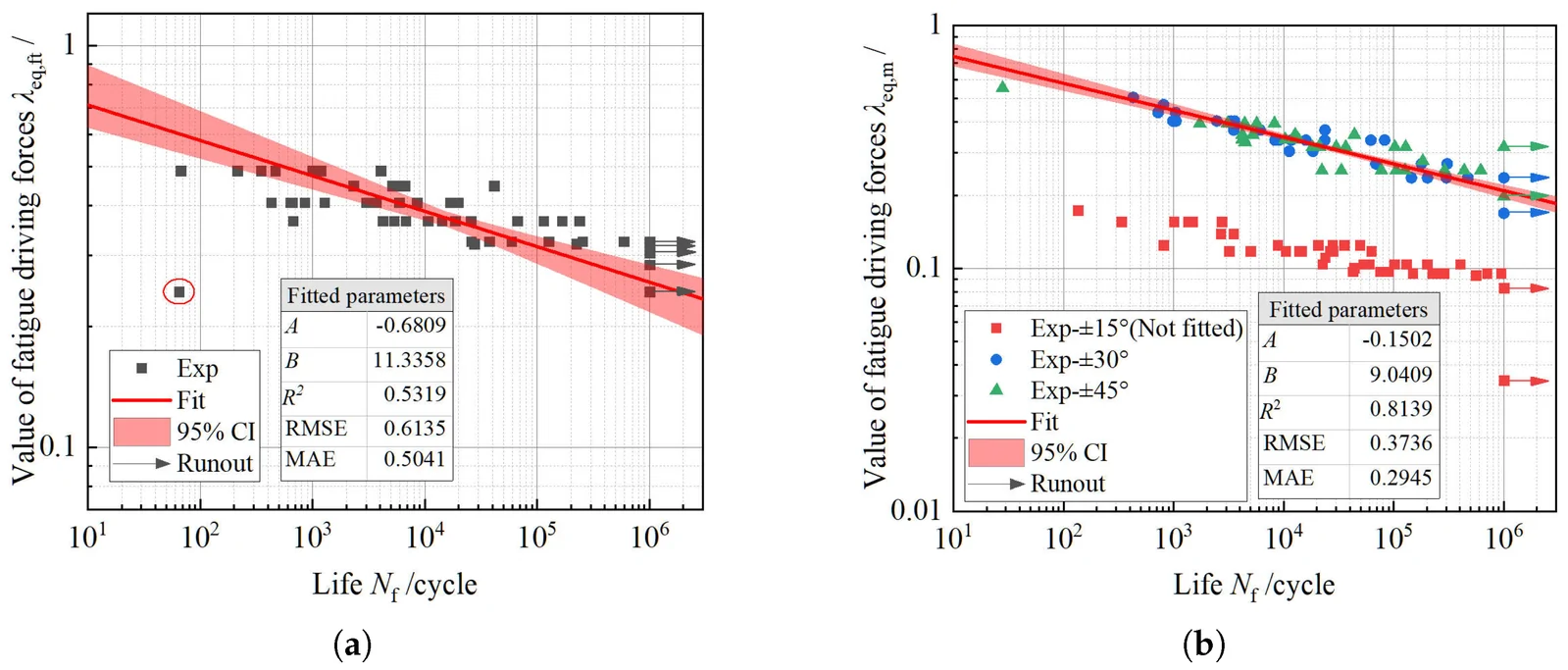
Fitted mode-specific S-N life relationships: (**a**) fiber-tension mode; (**b**) matrix-dominated mode.

Under the uniaxial tension–tension condition of the specimens at $R_{0}=0.1$, the fatigue-driving parameter varies proportionally with the cyclic load, such that $\lambda_{eq,i}=\lambda_{\max,i}$. The 0° fiber-tension data in Figure 7a exhibit appreciable scatter in life, and the relatively large number of runouts in the high-cycle regime broadens the confidence interval of the fit. The record marked by the red circle at 600 MPa and 65 cycles is inconsistent with the other results at the same stress level and was therefore excluded according to the above rule. In Figure 7b, the ±30° and ±45° data largely collapse onto a common trend in the $\lambda_{eq,m}$-$N_{f}$ space, indicating that the combined transverse-shear driving parameter provides a consistent representation of these two off-axis conditions. The ±15° data systematically deviate from this single matrix-dominated relation because of competition among the ft, *m*, and *s* modes and are therefore used only as a mixed-mechanism check.

The local material orientation, residual state after preloading, and valve-boss constraints cause the local fatigue-driving ratio at each integration point in the cylinder to differ from the specimen reference stress ratio. For each active mode, $\lambda_{\min,i}$ and $\lambda_{\max,i}$ are obtained from the low- and high-pressure equilibrium states, respectively, and the local fatigue-driving ratio, together with the amplitude and mean of the fatigue-driving parameter, are calculated from Equation (10):

(10) $$R_{\lambda ,i}=\frac{\lambda_{\min,i}}{\lambda_{\max,i}},\;\lambda_{a,i}=\frac{\lambda_{\max,i}-\lambda_{\min,i}}{2},\;\lambda_{\mathrm{mean},i}=\frac{\lambda_{\max,i}+\lambda_{\min,i}}{2}.$$

Hereafter, $R_{\lambda ,i}$ is referred to as the local fatigue-driving ratio. It is defined as the ratio of the nonnegative fatigue-driving parameters of the same mode at the low- and high-pressure states. This quantity is used to map the actual cyclic state at a cylinder integration point to the specimen reference stress ratio $R_{0}=0.1$; its numerical meaning differs from the conventional signed stress ratio $R_{\sigma }=\sigma_{\min}/\sigma_{\max}$. Within the tension-dominated range considered here, a dimensionless Goodman-type correction is used to obtain the equivalent maximum driving parameter at $R_{0}=0.1$ [29,51,53,54]:

(11) $$\lambda_{G,i}=\frac{\lambda_{a,i}}{1-\lambda_{\mathrm{mean},i}},\;a_{0}=\frac{1-R_{0}}{2},\;m_{0}=\frac{1+R_{0}}{2},\;\lambda_{eq,i}=\frac{\lambda_{G,i}}{a_{0}+\lambda_{G,i}m_{0}}.$$

The regular form of Equation (11) is evaluated when $\lambda_{\mathrm{mean},i}<0.999999$. In the UMAT, if $\lambda_{\mathrm{mean},i}$ reaches or exceeds 0.999999, the Goodman denominator is not evaluated; instead, $\lambda_{eq,i}$ is assigned a value of $1.0\times 10^{6}$. This numerical continuation rule avoids the Goodman singularity and provides a defined high equivalent driving parameter for the subsequent S-N evaluation. When the local fatigue-driving ratio $R_{\lambda ,i}$ equals $R_{0}$, the correction returns $\lambda_{eq,i}=\lambda_{\max,i}$, thereby preserving the specimen-calibration basis. Each material point then obtains $N_{f,ft}$ and $N_{f,m}$. The life-controlling mode under the current cyclic state can be identified from $\min(N_{f,ft},N_{f,m})$. However, because the controlling fatigue life also depends on the slope and intercept of Equation (9), it does not necessarily coincide with the peak static-strength mode having the largest $\lambda_{i}$.

The mode-specific S-N relationships determine the life scale of a material point under the current stress history. To track the progression of this calibrated life, $D_{i}\in \left[0,1\right]$ is introduced as the consumed life fraction; a separate variable $d_{i}$ maps this progression to the reduction in the corresponding load-carrying stiffness. Thus, $D_{i}$ quantifies the portion of the local fatigue life expended under the current cyclic stress history, whereas $d_{i}$ provides the direction-dependent stiffness feedback that updates the subsequent structural equilibrium. For $\Delta N_{k}$ cycles in the *k*th cycle block, the Miner-based update is

(12) $$\Delta D_{i,k}={\mathrm{ACTIVE}}_{i}\frac{\Delta N_{k}}{N_{f,i,k}},\;D_{i,k+1}=\min\left(1,D_{i,k}+\Delta D_{i,k}\right),\;D_{ctrl}=\max_{i\in \Omega }D_{i}.$$

Ω={ft,m} is the set of modes that have independent fatigue-test calibration and are actively used to advance life-fraction accumulation. ${\mathrm{ACTIVE}}_{i}=1$ for i∈Ω and ${\mathrm{ACTIVE}}_{i}=0$ otherwise. $N_{f,i,k}$ is the fatigue life of mode *i* evaluated from the local cyclic state at the start of cycle block *k*. The fiber-compression mode fc and independent shear mode *s* are retained as strength diagnostics and interfaces for future parameter extension. Fatigue-life progression in the present study is performed only through the ft and *m* modes supported by the tension–tension fatigue data. $D_{ctrl}=1$ marks the local fatigue endpoint of an integration point. For an integration point with $\lambda_{\mathrm{mean},i}\geq 0.999999$, the assigned $\lambda_{eq,i}$ produces a very short local fatigue life. The update in Equation (12) can then advance $D_{ctrl}$ to 1 and record the local fatigue endpoint.

A nonlinear power law is used to map life-fraction accumulation to stiffness degradation [27,55,56,57,58]:

(13) $$d_{f}=\min\left(D_{ft}^{p_{ft}},1-r_{E}\right),\;d_{m}=\min\left(D_{m}^{p_{m}},1-r_{E}\right),\;r_{E}=0.01.$$

where $D_{ft}$ and $D_{m}$ are the fiber-tension and matrix-dominated consumed life fractions, respectively; $d_{f}$ and $d_{m}$ are their corresponding stiffness-degradation variables; and $p_{ft}$ and $p_{m}$ are the calibrated power-law exponents. $r_{E}$ is the residual stiffness ratio. A value of $r_{E}=0.01$ is used, meaning that the affected modulus retains 1% of its initial value after the material point enters a highly damaged state. This avoids singularity of the degraded stiffness matrix and maintains numerical stability of the finite-element equilibrium iterations.

For the 0° specimens, the fiber-tension exponent was identified from the cyclic secant stiffness of stable hysteresis loops. The axial cyclic stiffness of the ±30° off-axis specimen cannot be equated directly with $E_{2}$ in the material-coordinate system; therefore, the local material-direction stiffness response was reconstructed using the actual lay-up and CLT before identifying the matrix-dominated exponent. The cyclic secant stiffness, specimen-level consumed life fraction, and normalized stiffness loss are calculated from Equation (14):

(14) $$K_{x,j}=\frac{\sigma_{x,\max,j}-\sigma_{x,\min,j}}{\varepsilon_{x,\max,j}-\varepsilon_{x,\min,j}},\;D_{j}=\frac{N_{j}}{N_{f}},\;{\hat{d}}_{j}=\frac{K_{x,0}-K_{x,j}}{K_{x,0}-K_{x,end}}.$$

where, $K_{x,j}$ is the axial cyclic secant stiffness of the *j*th stable hysteresis loop; $\sigma_{x,\max,j}$ and $\sigma_{x,\min,j}$, and $\varepsilon_{x,\max,j}$ and $\varepsilon_{x,\min,j}$ are its axial peak and valley stresses and strains, respectively. $N_{j}$ is the cycle count of that loop, $N_{f}$ is the measured specimen fatigue life, $D_{j}$ is the corresponding specimen-level consumed life fraction, $K_{x,0}$ is the stiffness of the first stable loop, $K_{x,end}$ is the stiffness of the final stable loop recorded before specimen failure, and ${\hat{d}}_{j}$ is the fitting target derived from the specimen secant stiffness. ${\hat{d}}_{j}$ is linked to the degradation variables in the material directions at an integration point either directly for the 0° case or through off-axis laminate-stiffness reconstruction; the axial stiffness of all off-axis specimens is therefore not incorrectly interpreted as a direct measurement of $E_{2}$.

**Figure 8:**
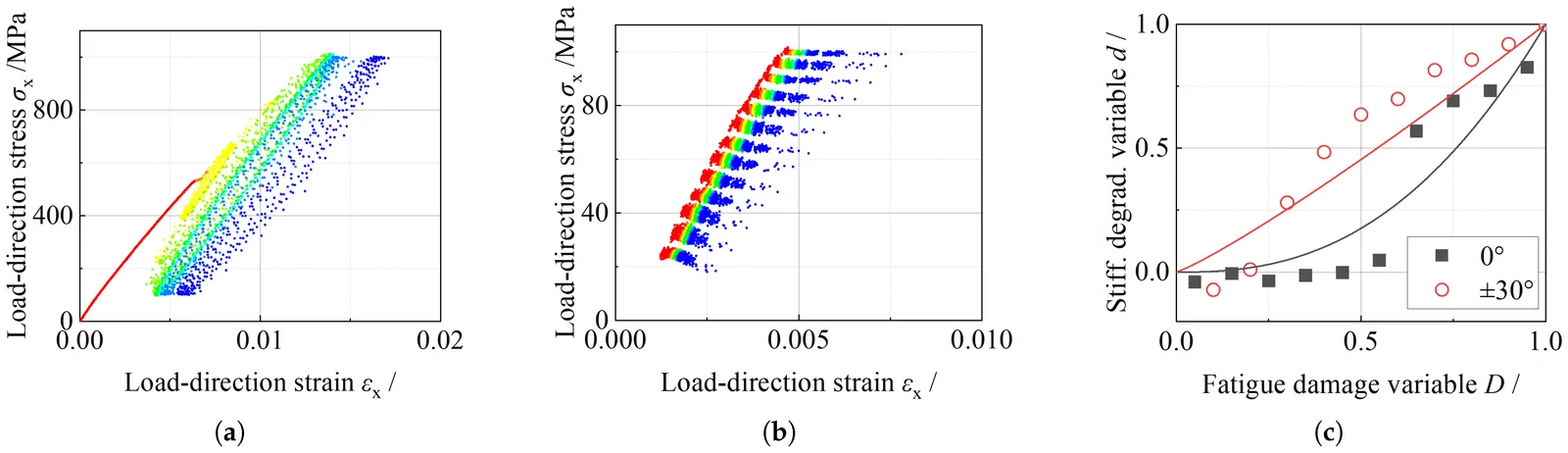
CFRP cyclic stress–strain data and identification of stiffness-degradation exponents: (**a**) cyclic stress–strain data for the 0° specimens; (**b**) cyclic stress–strain data for the ±30° specimens; (**c**) fitted relationship between normalized stiffness degradation and consumed life fraction for the 0° and ±30° specimens.

The fitted exponents are $p_{ft}=2.502$ and $p_{m}=1.138$. The fitting results are listed in Table 7, and Figure 8 illustrates the damage-stiffness-stress feedback relationship.

For the two degradation paths activated in the present model, the degraded orthotropic engineering constants are given by Equation (15):

(15) $$\begin{array}{cc} E_{1}^{d} & =\left(1-d_{f}\right)E_{1},\\ E_{2}^{d} & =\left(1-d_{m}\right)E_{2},\;E_{3}^{d}=\left(1-d_{m}\right)E_{3},\;G_{23}^{d}=\left(1-d_{m}\right)G_{23},\\ G_{12}^{d} & =G_{12},\;G_{13}^{d}=G_{13},\\ C^{d} & ={\left(S^{d}\right)}^{-1}. \end{array}$$

where the superscript *d* denotes a degraded quantity; $S^{d}$ and $C^{d}$ are the corresponding degraded compliance and stiffness matrices, respectively.

The stiffness-update scheme is defined according to the damage mechanisms governing the present hydraulic pressure cycling condition. The post-test burst morphology shows circumferential cracking and fiber-bundle fracture in the dome transition region near the equator, while the simulation identifies a connected fiber-tension failure region at the same location near the structural fatigue endpoint. These observations indicate that the final cylinder-level fatigue failure is governed primarily by the progressive loss of fiber-direction load-carrying capacity. Accordingly, the fiber-tension pathway is retained as the principal degradation mechanism for identifying the cylinder-level fatigue endpoint.

Shear-related effects are nevertheless included in the preceding damage evolution. The matrix-dominated fatigue-driving parameter contains the local in-plane shear stress $\tau_{12}$ together with the transverse normal stress; therefore, shear contributes to the evolution of $D_{m}$ and to the subsequent stress redistribution. The associated stiffness degradation is applied to $E_{2}$, $E_{3}$, and $G_{23}$, which characterize matrix-dominated deformation in directions normal to the fibers. By contrast, $G_{12}$ and $G_{13}$ describe shear deformation in planes containing the fiber direction. Applying $d_{m}$ directly to these constants would conflate the calibrated matrix-dominated degradation with an additional fiber-related shear-softening mechanism. $G_{12}$ and $G_{13}$ are therefore retained at their initial values in the present model, while the independent shear pathway remains available as an extension of the constitutive framework. This treatment retains the contribution of $\tau_{12}$ to fatigue evolution without obscuring the experimentally supported fiber-tension control of the final cylinder-level failure.

The direction-dependent stiffness changes in Equation (15) establish the intralaminar fatigue feedback used to update the CFRP response at integration points. The matrix-dominated reduction of $G_{23}$ characterizes the corresponding bulk stiffness response, whereas $G_{12}$ and $G_{13}$ are retained for the fiber-containing shear planes. This formulation provides the basis for analyzing the coupled progression of ply-level fatigue, stiffness degradation, stress redistribution, and the cylinder-level critical region. The local delamination observed after testing is considered together with resin cracking and fiber-bundle fracture as post-test evidence of the final failure morphology. An interface-resolved fatigue extension, for example, a fatigue cohesive-zone model calibrated with interface strength, mixed-mode fracture energy, and fatigue-propagation parameters, will provide the next step for quantifying interlaminar crack initiation and growth [59].

The major Poisson’s ratios remain fixed, the reciprocal Poisson’s ratios are recalculated from the reciprocity relations, and a symmetric three-dimensional compliance and stiffness matrix is then formed from the degraded engineering constants. The static strengths $X_{t}$, $X_{c}$, $Y_{t}$, $Y_{c}$, and $S_{12}$ remain fixed normalization references throughout cycling and are not degraded again with life-fraction accumulation, thereby avoiding double counting of the same fatigue damage through both strength reduction and S-N life progression.

In Figure 8a, the slope of the cyclic stress–strain response of the 0° specimens changes little during the early and middle portions of life and decreases markedly only near the end of life. This is consistent with fiber-direction load carrying remaining stable through most of the fatigue life and degrading rapidly at a late stage. By contrast, the cyclic strain of the ±30° off-axis specimens in Figure 8b expands progressively toward larger strain as life advances, indicating that transverse-shear coupled damage reduces the off-axis stiffness at an earlier stage. These differences produce the distinct degradation paths in Figure 8c: $p_{ft}=2.502>1$ concentrates fiber-direction degradation late in life, whereas $p_{m}=1.138$ causes matrix-dominated degradation to grow earlier and almost continuously with life-fraction accumulation. The two groups of tests therefore provide not only the degradation exponents but also the experimental basis for direction-dependent stiffness feedback in the finite-element model, in which transverse constraint is weakened first, and fiber load carrying is reduced more substantially at a later stage.

Solving the low- and peak-pressure equilibrium states cycle by cycle would make the computation of more than 20,000 hydraulic pressure cycles for the actual cylinder prohibitively expensive. A cycle-jump method constrained by the increment in consumed life fraction is therefore adopted. Within a cycle block, the current peak-valley stresses and fatigue-life-fraction accumulation rates are assumed to remain approximately constant, allowing repeated cycles with the same pressure range to be skipped. At the end of the block, the stiffness is updated, and a new equilibrium state is solved, rather than using the initial stress field throughout the entire life. Cycle jumping only groups repeated cycles and does not alter the pressure extrema or their sequence. This strategy is consistent with cycle-acceleration approaches used in progressive fatigue analysis [28,30].

Let the accumulated cycle count at the start of the current block be $N_{k}$, the block length be $\Delta N_{k}$, the maximum life-fraction increment per block be $\eta_{D}=0.02$, and the prescribed maximum cycle-block length be $\Delta N_{\mathrm{user}}$. By limiting $\Delta D_{i}$ for each mode participating in fatigue progression,

(16) $$\max_{i\in \Omega }\left(\frac{\Delta N_{k}}{N_{f,i,k}}\right)⩽\eta_{D},\;\Delta N_{k}=\min\left\{\Delta N_{\mathrm{user}},\left\lfloor \eta_{D}\min_{i\in \Omega }N_{f,i,k}\right\rfloor \right\}.$$

In Equation (16), $\Delta N_{\mathrm{user}}$ sets the maximum cycle-block length and controls computational efficiency. A value that is too small increases computational time, whereas a value that is too large reduces the accuracy of life progression. The parameter $\eta_{D}$ limits the life-fraction increment of each active mode within a single cycle block so that one jump consumes at most approximately $\eta_{D}$ of the local life, preventing the stiffness from degrading excessively within one block.

The calculation sequence for each cycle block is as follows:

1. Use $D_{ft,k}$ and $D_{m,k}$ at the start of the block to obtain $C_{k}^{d}$, solve equilibrium at the low and peak pressures, and store the local $\sigma_{11}$, $\sigma_{22}$, and $\tau_{12}$.
2. Calculate $\lambda_{\min,i}$ and $\lambda_{\max,i}$, check peak static-strength and mode-activation conditions, and obtain the local fatigue-driving ratio $R_{\lambda ,i}$ and $\lambda_{eq,i}$ from Equations (10) and (11).
3. Obtain $N_{f,ft}$ and $N_{f,m}$ from Equation (9), determine the cycle-block length from Equation (16), and advance $D_{ft}$ and $D_{m}$ according to Equation (12).
4. Update the degraded stiffness matrix using Equations (13)–(15), solve the structural equilibrium for the next cycle block, and update the accumulated cycle count to $N_{k+1}=N_{k}+\Delta N_{k}$. For P1, the internal variables of the three Maxwell branches are advanced over the physical time represented by the cycle block, $\Delta t_{k}=\Delta N_{k}T_{\mathrm{cyc}}$, where $T_{\mathrm{cyc}}$ denotes the prescribed duration of one complete hydraulic pressure cycle (30 s in the present tests).
5. If $D_{ctrl}$ crosses from below 1 to 1 or above within the current block, use local linear interpolation to locate the failure cycle of that integration point:

(17) $$N_{\mathrm{life},m}=N_{k}+\frac{1-D_{ctrl,k}}{D_{ctrl,k+1}-D_{ctrl,k}}\Delta N_{k}.$$

where $N_{\mathrm{life},m}$ is the interpolated local fatigue-endpoint cycle of material point *m*; the subscript *m* denotes the material-point label and is unrelated to the matrix-dominated mode. The cylinder-level predicted life, denoted hereafter by $N_{\mathrm{life}}$, is determined only when local endpoint regions become connected and peak-pressure equilibrium can no longer be re-established.

The constitutive model is implemented in Abaqus2020/Standard through a UMAT. In each cycle block, the current degraded stiffness is used to update the local stresses, mode-specific consumed life fractions, and failure flags, and the degraded stiffness matrix is returned to the finite-element solver as an approximate algorithmic tangent. The derivative contribution of within-block damage evolution to the tangent is not included.

When $D_{ctrl}$ reaches 1 within a cycle block, the local fatigue-endpoint cycle is first interpolated using Equation (17). The associated direction-dependent stiffness reduction is retained in the subsequent equilibrium solutions, thereby redistributing the hydraulic pressure load among the surrounding CFRP plies. At the cylinder level, the fatigue endpoint is identified only when neighboring local endpoint regions form a connected critical region and the finite-element model can no longer re-establish equilibrium under hydraulic pressure cycling. This criterion distinguishes an isolated local endpoint from cylinder-level structural termination.

The S-N and stiffness-degradation parameters above correspond to the carbon-fiber/epoxy specimens tested in this study at room temperature under tension–tension fatigue at $R_{0}=0.1$. Fatigue-life progression activates only the independently calibrated ft and *m* modes; fc and *s* are retained for peak-strength diagnostics and future extension.

## 5. Finite-Element Model and Loading History of the Type IV Cylinder

### 5.1. Structure, Lay-Up, and Boundary Conditions

**Figure 9:**
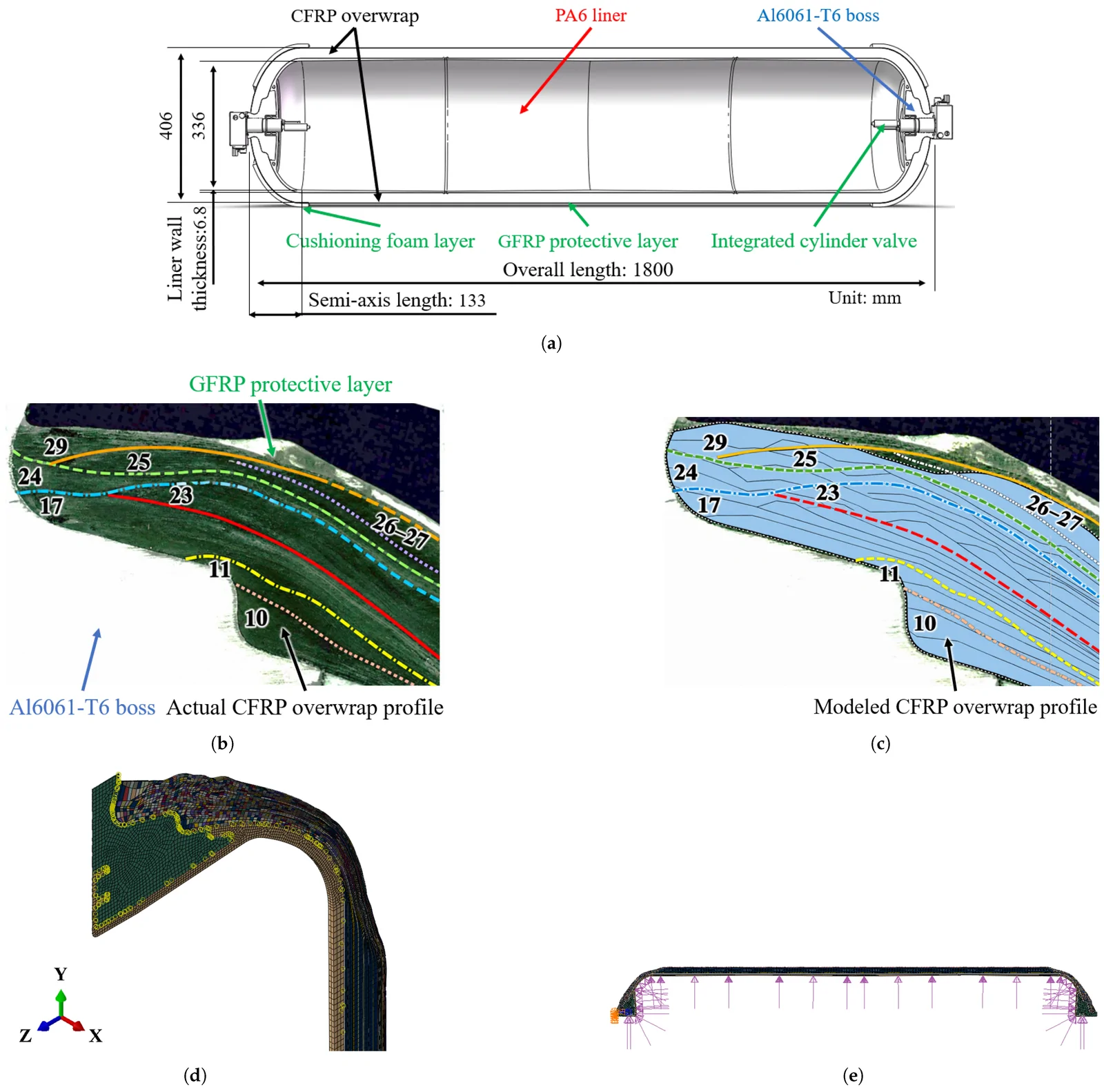
Finite element model of the V-1 cylinder: (**a**) cylinder structure and material components; (**b**) actual CFRP overwrap profile; (**c**) modeled CFRP overwrap profile; (**d**) finite-element mesh and Tie-interface locations; (**e**) internal-pressure loading and valve-boss constraints. In (**b**,**c**), colored contour lines trace selected CFRP overwrap ply boundaries and the adjacent numerals identify the ply numbers; in (**d**), yellow markers denote Tie-interface locations; and in (**e**), purple symbols denote the internal-pressure loading.

Previous studies have shown that the winding trajectory, fiber buildup thickness in the dome, stacking sequence, and circumferential ply-drop location jointly determine the local stress state and failure mode of composite pressure vessels [8,9,60,61]. Accordingly, a three-dimensional finite-element model of the V-1 cylinder was established, including the PA6 liner, a full CFRP overwrap, and two Al6061-T6 valve bosses, as shown in Figure 9a. Fiber buildup in the actual dome changes both the local thickness and material orientation and therefore cannot be replaced by a constant-thickness or idealized lay-up. The actual CFRP overwrap profile in Figure 9b was used to calibrate the dome geometry generated by the winding composite modeler 2017 (WCM2017). The winding model was adjusted until the modeled CFRP overwrap profile in Figure 9c matched the actual CFRP overwrap profile, thereby retaining the variations in curvature and ply orientation around the polar opening, dome transition, and regions adjacent to the valve bosses.

The nominal V-1 geometry had an outside diameter of 406 mm, an inside diameter of 336 mm, a PA6-liner thickness of 6.8 mm, an overall length of 1800 mm, and a dome semi-axis length of 133 mm. The CFRP overwrap was generated from the measured profile using 29 WCM winding rows, each defined by its winding angle, polar-opening radius, tape width, and ply thickness. The winding-angle sequence, in the order applied, was 90°, 75°, 67°, 90°, 24.2°, 24.1°, 90°, 25.5°, 90°, 24°, 24°, 90°, 16°, 90°, 12°, 90°, 14.2°, 18°, 21°, 26°, 30.2°, 35.3°, 18°, 13.9°, 18.9°, 29.6°, 29.6°, 35.3°, and 13°; the corresponding ply-thickness sequence was 1.88, 0.44, 0.44, 2.26, 0.65, 0.65, 2.26, 0.65, 2.26, 0.65, 0.65, 2.26, 0.44, 2.26, 0.44, 2.26, 0.44, 0.44, 0.44, 0.44, 0.44, 0.44, 0.44, 0.44, 0.44, 0.44, 0.44, 0.44, and 0.44 mm. WCM2017-generated local material orientations were retained for the cylinder-level fatigue calculation.

The PA6 liner was assigned either P0 or P1, the CFRP overwrap used the complete fatigue framework described in Section 4, and the two Al6061-T6 valve bosses were modeled using elastoplastic material parameters. Tie constraints were imposed between the liner and CFRP and between each valve boss and the adjacent liner/CFRP so that interface load transfer remained identical in the two controlled simulations. Because the fatigue-driving parameters are sensitive to dome curvature, regions adjacent to the interfaces, and through-thickness stress gradients, the mesh in Figure 9d was locally refined at the liner fillets, dome transition, and valve-boss connections. The nominal V-1 cylinder has rotationally periodic geometry, WCM2017-generated ply architecture, material distribution, internal-pressure loading, and valve-boss boundary conditions. Accordingly, the internal-pressure surface, valve-boss constraints, and periodic symmetry boundaries shown in Figure 9e were represented by a 1° circumferential sector. The periodic boundary conditions connect the two radial faces and allow this elementary sector to be rotationally arrayed to recover the complete cylinder, while retaining the local through-thickness and meridional stress variation needed for the cylinder-level fatigue analysis. Similar rotationally arrayed sector representations have been used in composite pressure-vessel finite-element analyses [62,63,64]. The material roles and key numerical settings are summarized in Table 8.

Mesh and cycle-block-length sensitivities were both evaluated using structural life as the response metric. With 32,239, 86,124, and 192,763 elements, the predicted lives were 19,205, 21,136, and 21,328 cycles, respectively, using the same P1 parameters and cycle-jump strategy. Taking the finest mesh as the reference, the 86,124-element mesh differed by only 0.90%, whereas the 32,239-element mesh differed by 9.95%; the 86,124-element mesh was therefore selected as the baseline. On this mesh, values of $\Delta N_{\mathrm{user}}$ of 1000, 300, and 150 cycles produced predicted lives of 16,675, 21,136, and 21,309 cycles, respectively. Relative to the 150-cycle result, the 300-cycle setting differed by 0.81%, whereas a value of 1000 cycles substantially underestimated life. A maximum cycle-block length of 300 cycles was therefore used in the subsequent calculations to balance computational cost and damage-progression accuracy.

### 5.2. Pressure History, Cycle Counting, and Output Metrics

**Figure 10:**
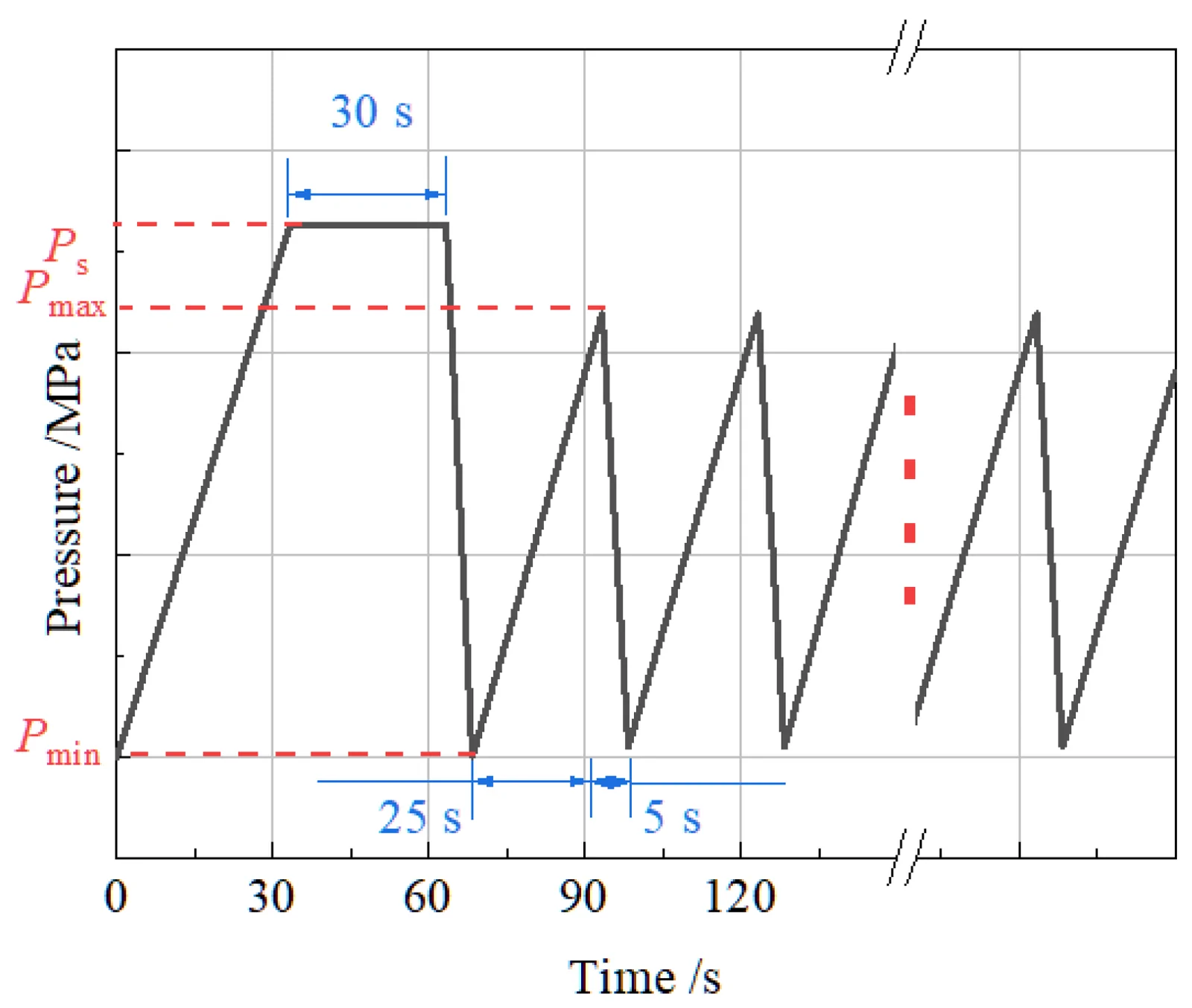
Loading history of the V-1 cylinder. The gray curve denotes the prescribed pressure history, the red dashed lines mark the labeled pressure levels, the blue arrows indicate segment durations, and the red ellipsis denotes omitted repeated cycles.

The finite-element model counts the factory hydrostatic preload separately from the subsequent fatigue cycles. As shown in Figure 10, pressure is first increased from 0 to 105 MPa at approximately 3.15 MPa/s, held for 30 s, and then unloaded. In accordance with the hydrostatic test requirement [65], this stage establishes residual plastic deformation in the PA6 and the initial local stress state in the CFRP but is not counted toward fatigue life. The 2–87.5 MPa cyclic pressure is applied only afterward [1,2]. This separation prevents the factory hydrostatic loading history from being incorrectly counted as service fatigue while retaining its influence on the initial residual state. For each cycle block containing $\Delta N_{k}$ cycles, CFRP life-fraction accumulation advances by $\Delta N_{k}$, while the viscoelastic internal variables in P1 advance over the corresponding physical time $\Delta N_{k}T_{\mathrm{cyc}}$. Equilibrium is resolved at the low- and peak-pressure endpoints, so cycle counting and time-dependent relaxation evolve synchronously under the same loading history.

Post-processing focuses on the time-dependent response of PA6, the local CFRP stresses $\sigma_{11}$, $\sigma_{22}$, and $\tau_{12}$, and the mode-specific consumed life fractions and stiffness-degradation variables. The structural endpoint is assessed from the formation of a continuous critical region together with the burst morphology. Except for the liner constitutive model, P0 and P1 share the same geometry, mesh, interfaces, CFRP parameters, and loading history, allowing differences in their results to be attributed specifically to the material model.

## 6. Results and Discussion

This section first compares the effects of P0 and P1 on the local CFRP fatigue state and predicted life, then traces cylinder-level failure along the evidence chain of fatigue-driving parameter-life-fraction accumulation-stiffness degradation-stress redistribution-critical-region connectivity, and finally discusses the engineering implications and applicability of the model.

### 6.1. Effect of the Time-Dependent PA6 Liner Response on the Local CFRP Fatigue State

Selection of the PA6 liner model should not be based solely on the quality of uniaxial curve fitting or the number of material parameters. Its effects on the CFRP fatigue-critical region, damage-evolution process, and predicted life should be compared directly, while also considering parameter-calibration effort and computational cost. Accordingly, controlled simulations were performed using the P0 elastoplastic model and the P1 elastoplastic–viscoelastic model under identical cylinder geometry, CFRP fatigue parameters, pressure history, mesh, and structural failure criterion. The only difference between the two simulations is the three-branch generalized Maxwell viscoelastic response introduced in P1. The comparison is summarized in Table 9.

**Figure 11:**
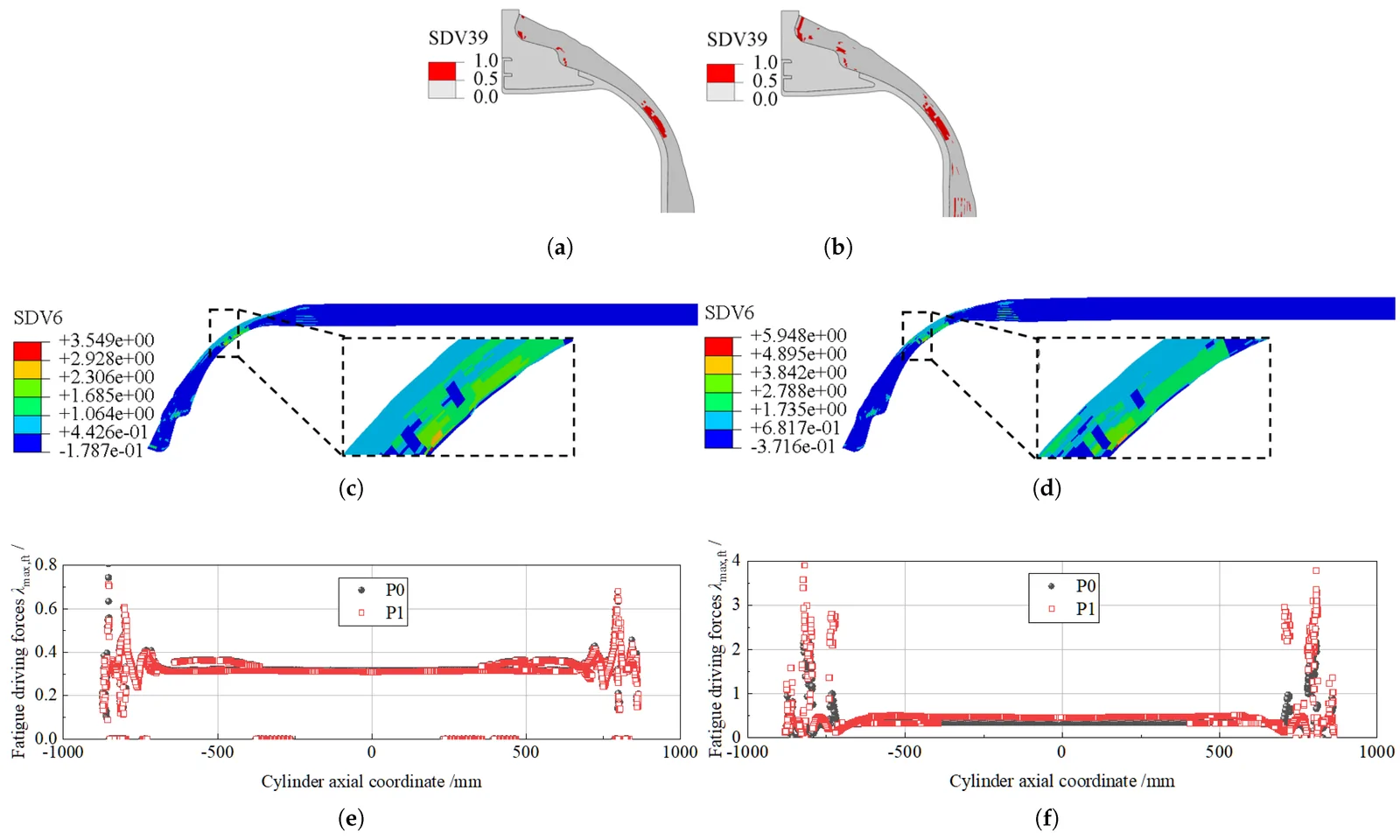
Comparison of CFRP fatigue response with the P0 and P1 liner models: (**a**) critical region at the P0 life endpoint; (**b**) critical region at the P1 life endpoint; (**c**) peak fiber-tension fatigue-driving parameter $\lambda_{\max,ft}$ at the P0 life endpoint; (**d**) peak fiber-tension fatigue-driving parameter $\lambda_{\max,ft}$ at the P1 life endpoint; (**e**) axial distribution of $\lambda_{\max,ft}$ at N=9000; (**f**) axial distribution of $\lambda_{\max,ft}$ at the life endpoint of each model.The black dashed boxes and connecting dashed lines identify the magnified dome regions shown in each panel.

As shown in Figure 11a,b, although P0 and P1 represent the time-dependent response of PA6 differently, both predict the final critical region in the dome transition region near the equator. This indicates that the spatial location of the critical region is governed primarily by dome curvature, ply angle, and the local load-transfer path; liner viscoelasticity mainly changes the evolving cyclic stress history at that location rather than moving the critical region to a different part of the structure. The difference is still small in the early stages of cycling. At N=9000, the axial distributions of $\lambda_{\max,ft}$ predicted by the two models in Figure 11e nearly coincide. As cycling proceeds, local stress differences caused by PA6 relaxation interact with CFRP life-fraction accumulation and stiffness degradation and accumulate progressively. Consequently, clear separation appears in the contour plots in Figure 11c,d and the axial distributions in Figure 11f at the respective life endpoints. It should be noted that the two endpoints correspond to different cycle counts and degradation stages; the endpoint maxima of 3.549 and 5.948 therefore represent two distinct damage-evolution paths reaching their respective termination states.

Table 9 further shows that this long-term accumulated difference ultimately appears in the predicted life. Relative to the mean burst life of the two burst-tested cylinders, the errors of P0 and P1 are 22.68% and 7.24%, respectively, while P1 requires 21.6% more computational time per cycle block than P0. P0 therefore remains suitable for rapid screening of the principal critical location, but neglecting recoverable relaxation causes fatigue damage to progress too rapidly for quantitative life prediction of the present cylinder. P1 provides a life prediction closer to the test result at a controlled increase in parameterization and computational cost and is therefore used as the liner baseline for the subsequent damage-evolution analysis.

Table 9 reports the computational time for one representative cycle block $\left(\Delta N_{k}=300\right)$, which directly compares the additional numerical cost introduced by the three Maxwell branches in P1. Relative to P0, P1 increases the representative per-block time by 21.6%. The completed P0 and P1 cylinder analyses required 59 and 73 cycle blocks, with total computational times of approximately $4.17\times 10^{5}$ s (116 h) and $6.27\times 10^{5}$ s (174 h), respectively. The total time therefore reflects both the per-block constitutive-update cost and the number of blocks required to reach the respective predicted fatigue endpoint; the representative per-block time provides the more direct comparison of model complexity.

Accordingly, P1 is used as the baseline liner model for all subsequent cylinder-level damage-evolution analyses. The remaining question is how spatially different local fatigue-driving parameters evolve, through life-fraction accumulation, stiffness degradation, and stress redistribution, into a continuous critical region that controls cylinder life.

### 6.2. Development of Local CFRP Fatigue Damage into Structural Failure

The local stress state at the beginning of cycling determines the magnitude of the fatigue-driving parameters and can identify only the propensity for damage initiation. The final critical region also depends on stiffness degradation and stress redistribution after damage. The P1 baseline simulation is therefore traced sequentially through fatigue-driving parameters, life-fraction accumulation, direction-dependent stiffness degradation, stress redistribution, and connectivity of the critical region.

**Figure 12:**
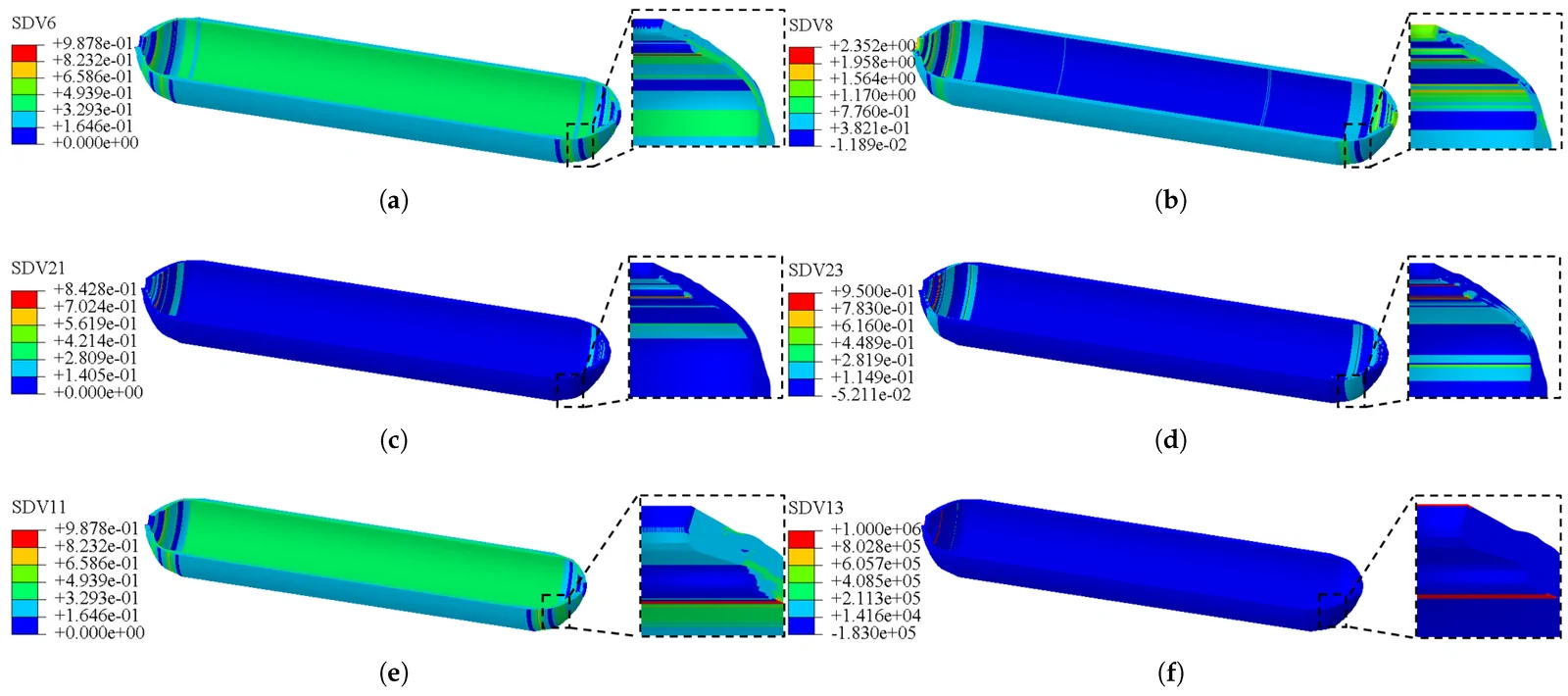
Spatial distributions of fatigue-driving parameters in the CFRP overwrap: (**a**) peak fiber-tension fatigue-driving parameter $\lambda_{\max,ft}$; (**b**) peak matrix-dominated fatigue-driving parameter $\lambda_{\max,m}$; (**c**) local fatigue-driving ratio $R_{\lambda ,ft}$ for the fiber-tension mode; (**d**) local fatigue-driving ratio $R_{\lambda ,m}$ for the matrix-dominated mode; (**e**) equivalent fiber-tension fatigue-driving parameter $\lambda_{eq,ft}$; (**f**) equivalent matrix-dominated fatigue-driving parameter $\lambda_{eq,m}$.

The peak fatigue-driving parameters in Figure 12a,b are strongly nonuniform across the dome, polar opening, and plies adjacent to the valve boss because winding angle, curvature, thickness, and end constraints jointly affect the local stress state. More importantly, the low- and high-pressure states do not vary by the same proportion at all integration points. As shown in Figure 12c,d, $R_{\lambda ,ft}$ and $R_{\lambda ,m}$ vary substantially between the cylindrical section, dome, and different through-thickness positions; the externally imposed nominal pressure ratio therefore cannot be used directly as a substitute for the local fatigue-driving ratio. After the Goodman-type correction, Figure 12e,f map these local cyclic states to the common specimen reference stress ratio $R_{0}=0.1$, thereby changing the equivalent driving level with which different locations enter the S-N relationship. Figures 12e,f show that the outer plies near the polar opening and the inner plies adjacent to the valve-boss step exhibit relatively high matrix-dominated and fiber-tension equivalent driving parameters, respectively, indicating that these locations tend to consume local life first.

**Figure 13:**
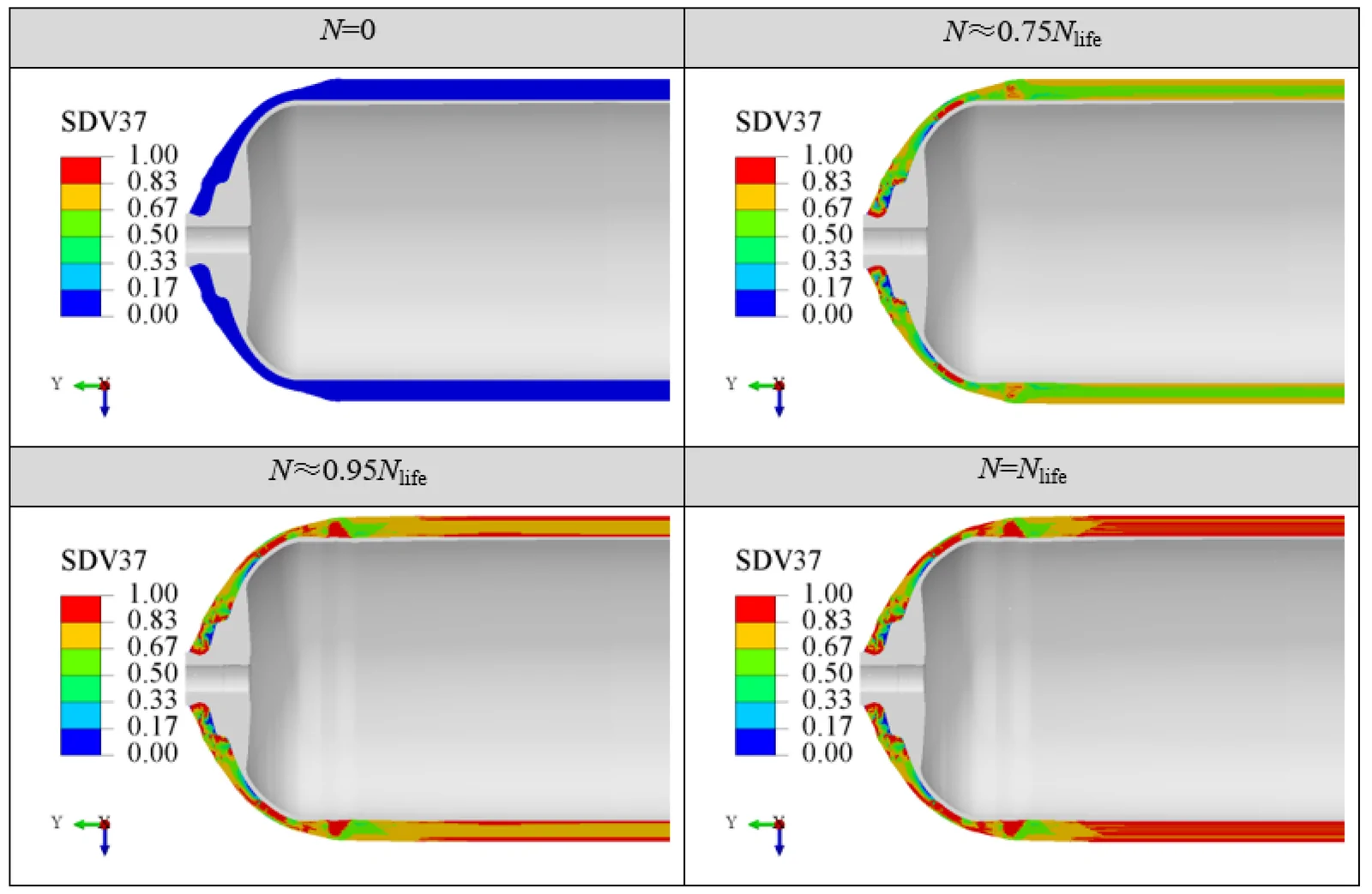
Spatial propagation of the CFRP consumed life fraction.

Figure 13 explains why a point with a high initial fatigue-driving parameter does not necessarily coincide with the final structural critical region. At N=0, preloading establishes only the residual stress–strain state, and no fatigue life has yet been consumed. By $N\approx 0.75N_{\mathrm{life}}$, discrete regions of high $D_{\mathrm{ctrl}}$ first appear in the dome transition, but the surrounding plies, which have not yet degraded substantially, can still share the load; the local high values therefore do not yet trigger structural termination. As stiffness decreases in these regions, load is transferred to adjacent plies, increasing their subsequent rate of life-fraction accumulation. The highly damaged region consequently continues to expand along the dome-cylinder transition at $N\approx 0.95N_{\mathrm{life}}$, and a continuous high-damage band near the equator forms only at $N=N_{\mathrm{life}}$. This evolution shows that the final critical region is the spatial outcome of the feedback sequence local damage-stiffness reduction-load redistribution-additional damage in neighboring regions, rather than simply the location of the initial contour maximum.

**Figure 14:**
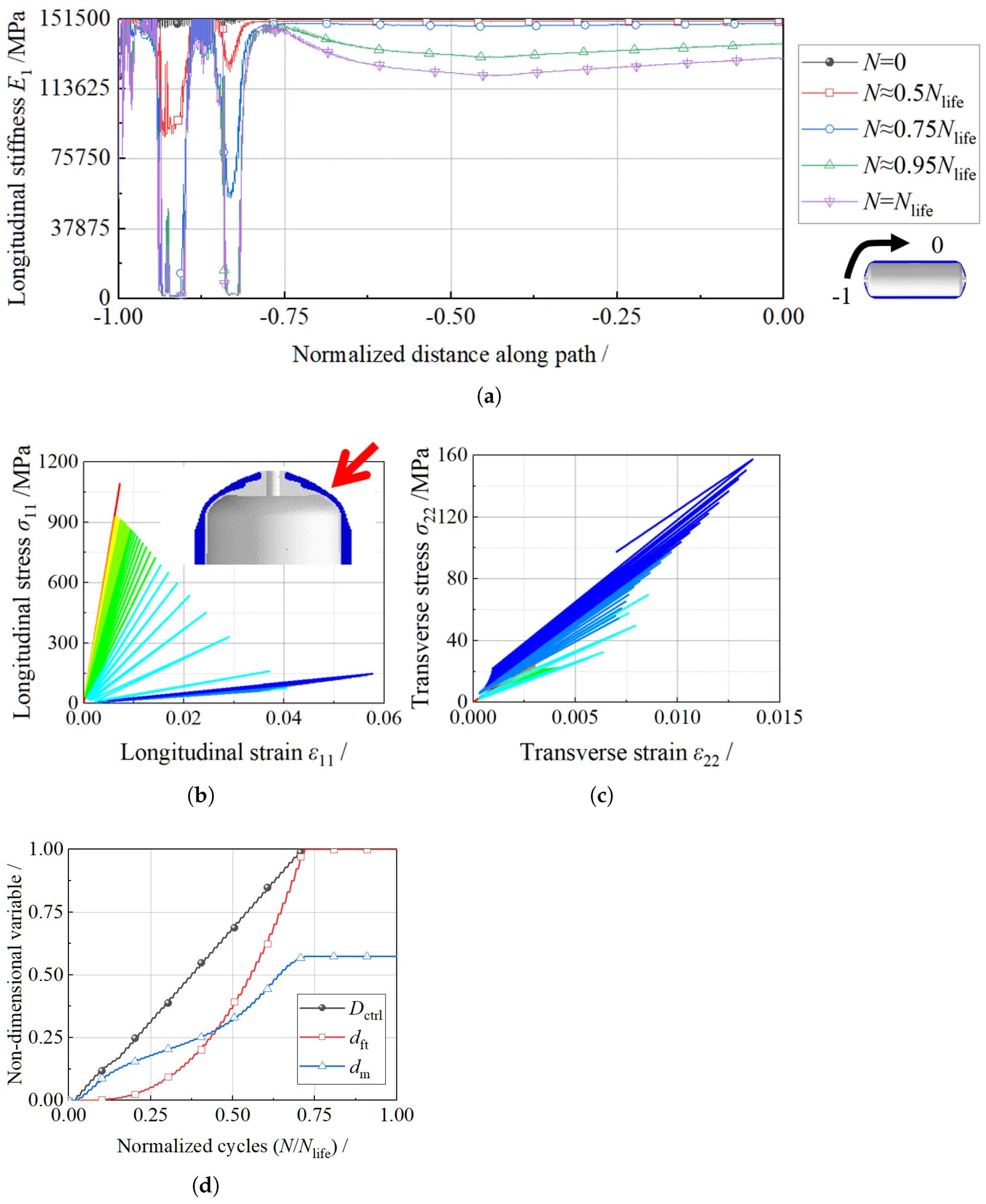
Direction-dependent stiffness degradation in the CFRP overwrap: (**a**) overall stiffness evolution of critical ply 11; (**b**) cyclic fiber-direction stress–strain response at a critical material point in the dome; (**c**) cyclic transverse matrix stress–strain response at a critical material point in the dome; (**d**) evolution of the consumed life fraction and stiffness-degradation variable at the critical material point. In (**a**–**c**), colors distinguish responses extracted at successive cycle stages, progressing from the initial state to $N=N_{\mathrm{life}}$.

Figure 14 further explains the spatial expansion in Figure 13 through the evolution of local stiffness. Ply 11 lies on a principal load-transfer path in the dome. As shown in Figure 14a, its fiber-direction stiffness does not decrease uniformly throughout the ply; instead, a degraded region first develops locally in the dome. At $N=N_{\mathrm{life}}$, the local stiffness in the dome has fallen to nearly zero, whereas the cylindrical section still retains a high stiffness, reflecting spatial differences in consumed life. Figure 14b shows that, as the number of cycles increases, the cyclic fiber-direction stress–strain response at the critical dome material point progressively expands toward larger strain and its slope decreases, indicating degradation of the effective fiber-direction stiffness. The transverse response in Figure 14c likewise exhibits a lower slope and a wider transverse strain range, showing that matrix-dominated damage progressively reduces the transverse stiffness of the material point and alters the local deformation response of adjacent plies. These two forms of direction-dependent stiffness degradation provide the mechanical basis for the subsequent stress redistribution.

The sequence of stiffness degradation is clear in Figure 14d. The matrix-dominated stiffness degradation variable, $d_{m}$, increases continuously from the early stage of cycling, whereas the fiber-tension stiffness degradation variable $d_{f}$ surpasses $d_{m}$ after approximately $0.35N_{\mathrm{life}}$, enters an accelerated growth stage, and reaches unity at approximately $N\approx 0.70N_{\mathrm{life}}$, when the local termination criterion $D_{\mathrm{ctrl}}=1$ is satisfied. At this point, the cylinder-level fatigue-life progression has reached only approximately $0.70N_{\mathrm{life}}$; structural fatigue failure has not yet occurred, and the full cylinder remains capable of sustaining subsequent hydraulic pressure cycles. This is because adjacent plies retain residual stiffness and share part of the load after failure of an individual material point. Thus, early matrix degradation modifies the local constraint, rapid late-stage fiber degradation triggers local failure, and load transfer to neighboring plies determines whether that local failure develops further into a continuous cylinder-level critical region.

**Figure 15:**
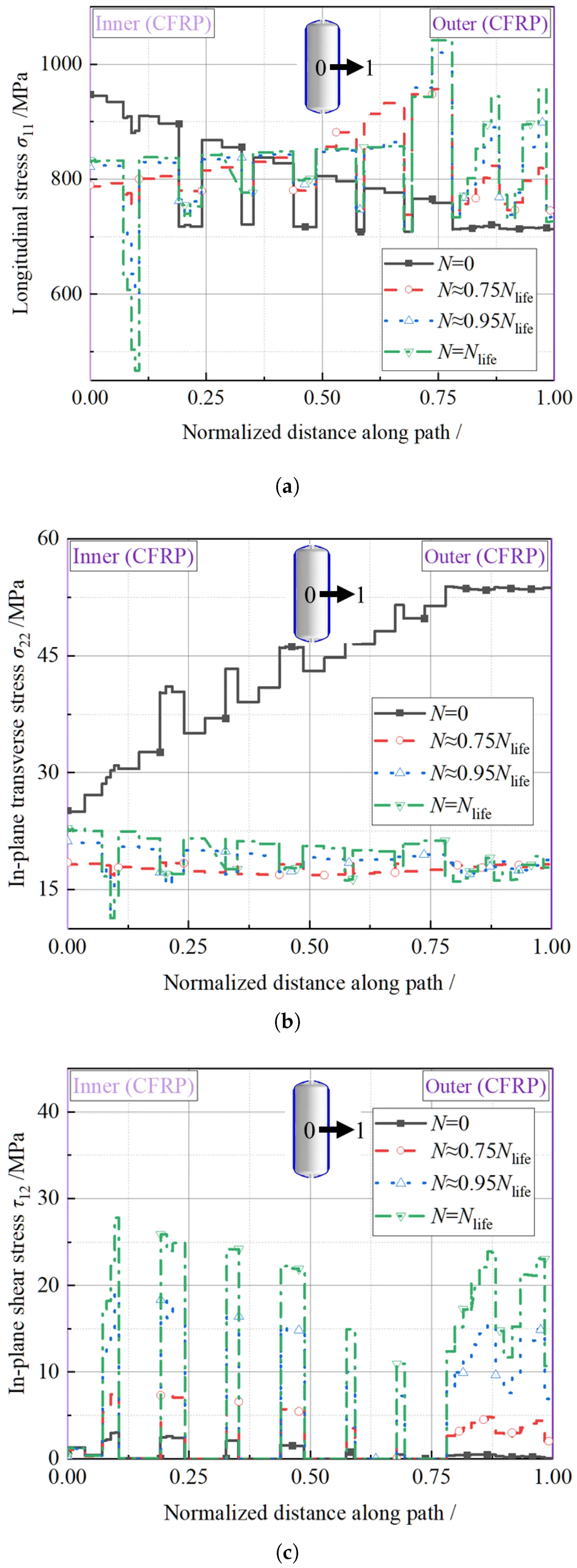
Through-thickness stress redistribution in the CFRP: (**a**) fiber-direction stress $\sigma_{11}$; (**b**) transverse stress $\sigma_{22}$; (**c**) in-plane shear stress $\tau_{12}$.

Stiffness feedback changes the through-thickness load-sharing pattern. As shown in Figure 15a, the fiber-direction stress $\sigma_{11}$ is carried more heavily by the inner plies early in cycling. Once the inner plies undergo fiber-direction stiffness degradation, their load share decreases and the outer plies, which are less degraded, carry a higher $\sigma_{11}$ after structural re-equilibration, producing an outward transfer of fiber-direction load. In Figure 15b, $\sigma_{22}$ generally decreases with cycling because matrix-dominated damage reduces $E_{2}/E_{3}$ and the associated transverse constraint, making it difficult for degraded plies to maintain their initial transverse stress level. Meanwhile, $\tau_{12}$ in the 90° hoop plies remains close to zero in Figure 15c, whereas $\tau_{12}$ in the helical plies gradually increases from near zero, indicating increased participation of in-plane shear as the longitudinal and transverse load components are redistributed. The three stress components therefore do not degrade synchronously; together, they reflect load reconstruction caused by direction-dependent stiffness reduction. Representative normalized changes are listed in Table 10.

**Figure 16:**
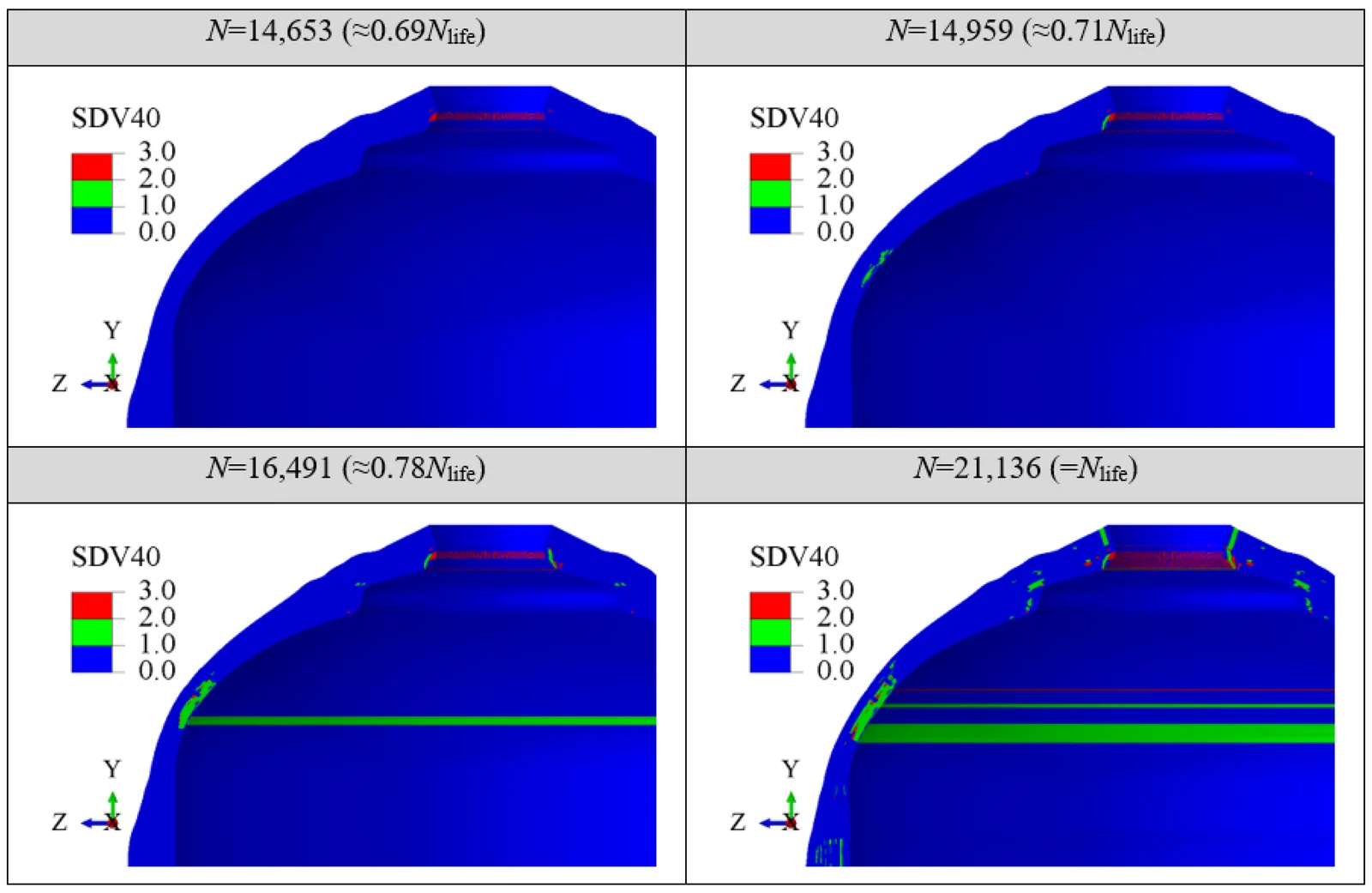
Formation of mode-specific local CFRP failure and the continuous critical region. (SDV40 is the local failure-mode identifier: 0 = unfailed, 1 = fiber-tension failure, and 3 = matrix-dominated failure.)

Figure 16 further shows how load transfer changes the spatial organization of local failure modes. At approximately $0.69N_{\mathrm{life}}$, matrix-dominated failure has already appeared in local regions with high-driving parameters. Fiber-tension failures then increase progressively, consistent with the temporal sequence in Figure 14d, in which matrix degradation occurs first and fiber degradation accelerates later. Not all initially high-driving points near the polar opening or valve-boss step evolve into the final structural endpoint. After local stiffness decreases, these locations unload and part of the load is transferred to adjacent plies that have not yet failed. As cycling continues, failed elements in the dome transition region near the equator progressively expand and become connected. This combination of modes is compatible with the coexistence of resin cracking, local delamination, and fiber-bundle fracture observed after testing in Figure 2c,d, although the post-test photographs are not used to infer the temporal order of individual failure modes.

According to the fatigue-failure criterion in Section 4, $D_{ctrl}=1$ at a single integration point represents only local fatigue termination. The CFRP load-bearing structure of the cylinder is considered to have failed only when the failed region further connects into a critical band in the dome and the peak-pressure step can no longer re-establish equilibrium. Consequently, the life comparison in Table 11 uses only burst-tested cylinders #1 and #2, whose termination mechanisms are consistent with this criterion. Their mean life of 22,785 cycles is used as the structural burst-life reference. The valve leakage of cylinder #3 is retained to represent the competing leakage-before-burst termination mechanism and the applicability limits of the model.

**Figure 17:**
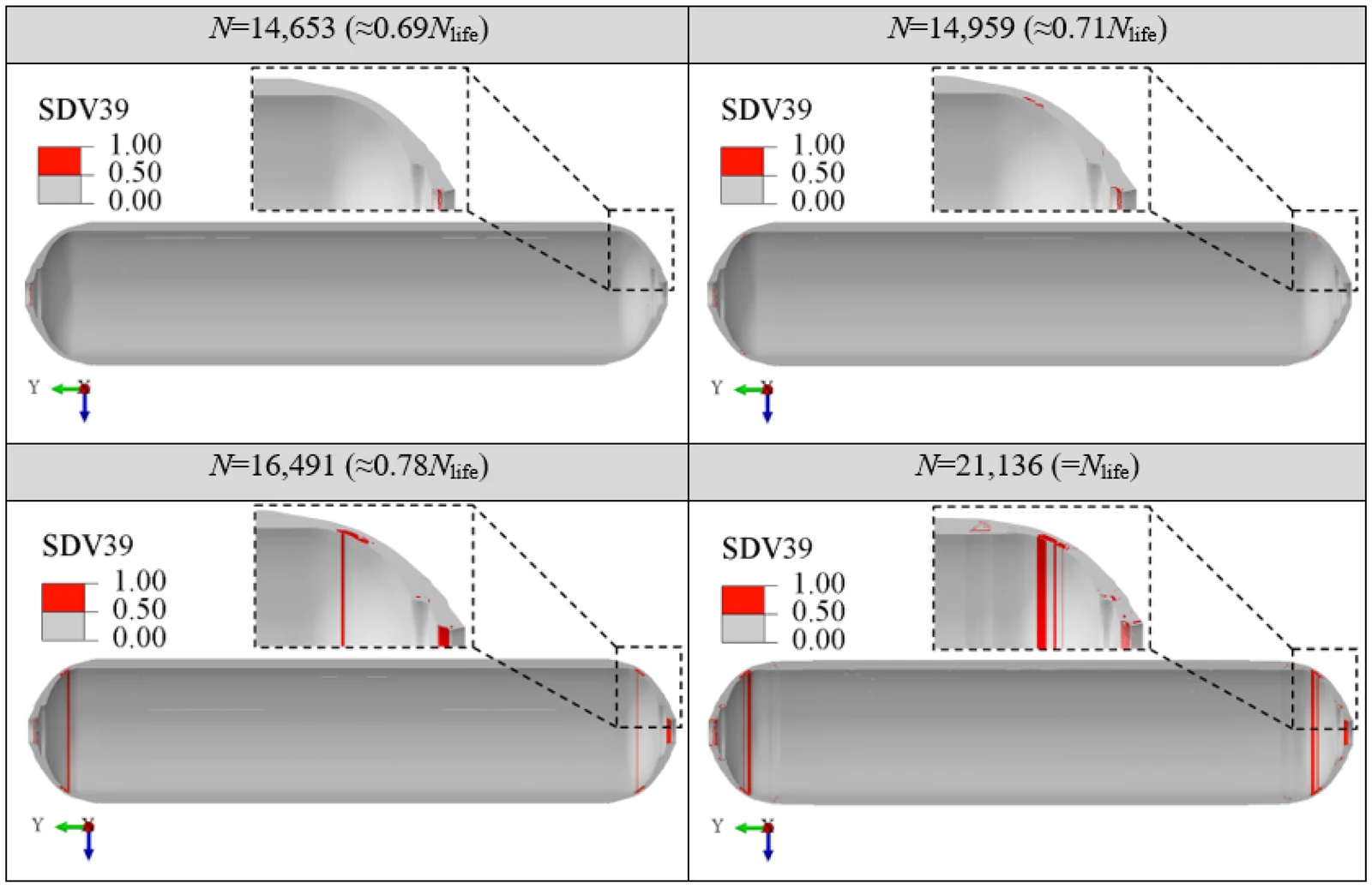
Propagation of the local CFRP failure region predicted by P1. (SDV39 is the local failure flag: 0 = unfailed and 1 = failed.)

The P1 prediction of 21,136 cycles is lower than the measured burst lives of 22,357 and 23,213 cycles, providing a conservative estimate of the structural burst life for the present cylinder. Figure 17 explains why earlier local failure does not immediately imply termination of the full cylinder. Between $N\approx 0.69N_{\mathrm{life}}$ and $0.71N_{\mathrm{life}}$, the local failure flag SDV39 indicates only local failure in the dome transition, while surrounding plies continue to maintain overall equilibrium through load transfer. After approximately $N\approx 0.78N_{\mathrm{life}}$, adjacent regions continue to fail under the redistributed load, ultimately forming a connected failure band in the dome transition region near the equator at $N=N_{\mathrm{life}}$. This location corresponds to the circumferential crack-initiation region observed in both burst-tested cylinders in Figure 2c,d, showing that the model predicts not only the life scale but also the spatial location controlling structural failure.

Thus, the life values in Table 11, the correspondence between the critical region in Figure 2c,d and Figure 17, and the compatibility between the mode patterns in Figure 16 and the post-test multi-mechanism failure morphology together form a cylinder-level evidence chain at the life, spatial, and failure-mechanism levels. The post-test morphology is used only to support mode compatibility and not to establish the temporal sequence predicted numerically.

### 6.3. Engineering Implications and Model Applicability

An important limitation is that the same two burst-tested cylinders are used both for the P0/P1 structural comparison and for the final life comparison. The 7.24% deviation therefore represents in-sample agreement for the present cylinder configuration and pressure history rather than a generalization error evaluated on an independent external sample. From an engineering perspective, P0 can be used for rapid screening of critical locations. When quantitative life prediction is required, P1 is more appropriate for the present loading condition because, although PA6 is not the primary pressure-bearing layer, its time-dependent response still changes the long-term cyclic stress input to the CFRP. Similarly, the local fatigue-driving ratio in the actual overwrap varies with ply angle, through-thickness position, and structural constraint and cannot be replaced by a single externally imposed nominal pressure ratio. The critical region should also emerge from the damage-stiffness-stress feedback process rather than being assigned directly from a single high-stress point in the initial cycle.

The parameter basis of the present study is limited to PA6 at approximately 3.59 wt.% moisture uptake, an ambient temperature of 23 °C, CFRP tension–tension fatigue data at $R_{0}=0.1$, and the actual V-1 winding architecture. Other local fatigue-driving ratios are mapped through the Goodman-type relationship, and the structural loading path is hydraulic pressure cycling between 2 and 87.5 MPa. The cycle-jump procedure advances CFRP damage and the viscoelastic internal variables of PA6 synchronously with the corresponding physical time; however, the present framework evaluates only intralaminar fatigue of the CFRP and does not include competing failure mechanisms such as valve-seal leakage. Within the present scope, the post-test local delamination complements the observed resin cracking and fiber-bundle fracture in characterizing the final failure morphology, while the numerical analysis resolves the associated intralaminar fatigue progression and cylinder-level structural response. Because P0 and P1 use the same interface treatment, this scope does not affect their controlled comparison. A subsequent interface-resolved extension with independently calibrated interface properties will enable fatigue-delamination mechanisms to be incorporated into the cylinder-level framework. Application to other moisture conditions, temperatures, material systems, cylinder configurations, or loading histories requires recalibration of the material parameters and validation of the cylinder-level termination criterion.

## 7. Conclusions

(1) PA6 elastoplastic model P0 and elastoplastic–viscoelastic model P1 were established for a moisture condition of 3.59 ± 0.16 wt.%, close to that of the actual liner. P1 uses a three-branch generalized Maxwell network to represent the 6 h stress–relaxation response, with a fit of $R^{2}=0.97$, thereby adding the recoverable time-dependent response that cannot be represented by P0.
(2) A mode-specific progressive fatigue framework for CFRP was established in the material-coordinate system. The 0° specimens were used to calibrate the fiber-tension (ft) life relationship, while the ±30° and ±45° specimens were used to calibrate the matrix-dominated (*m*) relationship. Local fatigue-driving-ratio correction, mode-specific S-N relationships, Miner-based life-fraction accumulation, direction-dependent stiffness degradation, and cycle jumping together form a closed-loop update at the integration-point level.
(3) Relative to the mean measured life of 22,785 cycles for the two burst-tested cylinders, P0 and P1 predicted 17,617 and 21,136 cycles, corresponding to errors of 22.68% and 7.24%, respectively. With identical geometry, CFRP parameters, and loading history, P1 substantially improved agreement with the measured life at the cost of a 21.6% increase in computational time. This indicates that PA6 viscoelasticity should be retained for quantitative life analysis under the material condition and hydraulic loading path investigated here.
(4) The P1 results show that matrix-dominated damage first weakens the transverse constraint, followed by a rapid reduction in fiber-direction stiffness. This causes $\sigma_{11}$ to shift from the inner to the outer plies, $\sigma_{22}$ to decrease, and $\tau_{12}$ to increase in some helical plies, ultimately forming a continuous failure band in the dome transition region near the equator. Its location corresponds to the circumferential crack region observed in both burst-tested cylinders. The third cylinder developed valve leakage at 20,005 cycles, demonstrating that the present model covers only the CFRP structural fatigue endpoint and does not include seal failure. The conclusions are therefore limited to the current material system, moisture condition, actual winding architecture, and 2–87.5 MPa hydraulic pressure cycling path.

## Figures and Tables

**Table 1:** CFRP specimen types and parameter-calibration roles.

Specimen Type	Lay-up or Dimensions	Purpose
0° tension and tension–tension fatigue	[0_4_]; 230 × 12.5 mm; thickness ≈ 1.3 mm	Calibrate fiber-direction stiffness and fiber-tension fatigue
±15°/±30°/±45° fatigue	[+θ_4_/−θ_4_]; 180 × 25 mm; thickness ≈ 2.6 mm	Identify off-axis combined stresses and dominant modes
90° tension	Transverse specimens cut from a 12-ply unidirectional laminate	Determine transverse elastic and tensile properties
V-notched beam shear	76 × 20 mm; thickness ≈ 4.0 mm	Determine $G_{12}$ and $S_{12}$
NOL ring specimen	Diameter ≈ 150 mm; thickness ≈ 1.5 mm	Determine fiber-direction tensile strength under a state close to filament winding

**Table 2:** Complete hydraulic pressure cycling termination records for the V-1 cylinders.

Cylinder	Pressure Range/MPa	Termination Cycle	Termination Mode	Main Post-Test Observations
V-1-#1	2–87.5	23,213	Burst	Fiber fracture, resin cracking, local delamination; circumferential cracking in the dome transition region near the equator
V-1-#2	2–87.5	22,357	Burst	Fiber fracture, resin cracking, local delamination; circumferential cracking in the dome transition region near the equator
V-1-#3	2–87.5	20,005	Leakage before burst	Water leakage at the cylinder valve and observed resin cracking

**Table 3:** PA6 elastoplastic parameters and experimental results under two stress–strain measures.

Parameter	Engineering Stress–Strain Result	True Stress–Logarithmic Strain Result	Value Used in P0/P1
Elastic modulus *E*/MPa	973.79	978.21	978.21
0.2% offset yield strength $\sigma_{y}$/MPa	28.84	29.76	29.76
Linear hardening modulus *H*/MPa	5.32	41.41	41.41
Ultimate strength $\sigma_{u}$/MPa	36.96	101.73	Used for checking experimental features
Poisson’s ratio ν	0.35	0.35	0.35

**Table 4:** Calibration results for the three-branch generalized Maxwell model of PA6 over the 6 h relaxation window.

Quantity	Parameter Definition	Value	Unit
$E_{\infty }$	Long-term modulus from stress–relaxation fit	510.64	MPa
$E_{1}$/$\tau_{1}$	Short-time branch	204.66/7.79	MPa/s
$E_{2}$/$\tau_{2}$	Intermediate-time branch	107.73/365.64	MPa/s
$E_{3}$/$\tau_{3}$	Long-time branch	113.31/9437.55	MPa/s
$E_{0,\mathrm{fit}}$	Initial modulus from relaxation fit (sum of all terms)	936.34	MPa
$E_{\mathrm{eq}}$	Three-dimensional equilibrium-network modulus used in P1	552.51	MPa
$R^{2}$/RMSE/MAE	Fit quality over the 6 h relaxation window	0.97/14.90/11.38	—/MPa/MPa

**Table 5:** Orthotropic elastic and intralaminar static-strength parameters of CFRP.

Parameter Group	Parameter	Value	Unit	Source
Elastic	$E_{1}$/$E_{2}$/$E_{3}$	151,500/8830/8830	MPa	0° and 90° tensile tests
Elastic	$G_{12}$/$G_{13}$	5070/5070	MPa	V-notched beam shear test
Elastic	$G_{23}$	3100	MPa	Ref. [39]
Elastic	$\nu_{12}$/$\nu_{13}$	0.307/0.307	—	0° tensile test
Elastic	$\nu_{23}$	0.38	—	Ref. [39]
Strength	$X_{t}$	2461.794	MPa	NOL ring test
Strength	$X_{c}$	1470	MPa	Ref. [39]
Strength	$Y_{t}$/$Y_{c}$	71/1030	MPa	Ref. [39]
Strength	$S_{12}$	63.8	MPa	V-notched beam shear test

**Table 6:** S-N fitting parameters for the fiber-tension and matrix-dominated modes (reference stress ratio $R_{0}=0.1$).

Dominant Mode	Number of Data Points *n*	$A_{i}$	$B_{i}$	$R^{2}$	RMSE (Log Life)	MAE (Log Life)
Fiber-tension ft	47	−0.6809	11.3358	0.5319	0.6135	0.5041
Matrix-dominated *m*	60	−0.1502	9.0409	0.8139	0.3736	0.2945

**Table 7:** Degradation exponents, fit quality, and modulus-feedback targets for the active fatigue modes.

Mode	Life Variable	Degradation Variable	Power Exponent *p*	$R^{2}$	RMSE	MAE	Primary Modulus Feedback
Fiber-tension ft	$D_{ft}$	$d_{f}$	2.502	0.7073	0.2027	0.1527	$E_{1}$
Matrix-dominated *m*	$D_{m}$	$d_{m}$	1.138	0.6091	0.3097	0.2580	$E_{2}$, $E_{3}$, $G_{23}$
Local control	$D_{ctrl}=\max\left(D_{ft},D_{m}\right)$	—	—	—	—	—	Failure flag and spatial-connectivity analysis

**Table 8:** Material roles and key numerical settings of the V-1 model.

Component or Setting	Model/Value	Purpose
PA6 liner	P0 or P1 (controlled comparison); P1 used thereafter	Liner deformation, pressure hold/unloading, and load transfer
CFRP overwrap	Three-dimensional orthotropic elasticity + mode-specific progressive fatigue	Structural load carrying, fatigue life-fraction accumulation, and stiffness feedback
Al6061-T6 valve bosses	Elastoplastic: E=68,000 MPa, ν=0.33, $\sigma_{y}=270$ MPa, $\epsilon_{f}=14.8\%$ (elongation at fracture), $\sigma_{u}=307$ MPa,	Connection and local end constraint
Computational domain	1° circumferential sector	Reduce computational cost using structural periodicity
Mesh	Local refinement in regions of high curvature and connection	Resolve local stresses and through-thickness gradients
Numerical sensitivity	Mesh density and maximum cycle-block length	Assess sensitivity of life prediction to numerical discretization and determine the baseline scheme
Loading history	0 → 105 MPa, hold for 30 s, then unload	Establish the residual state before cycling; not counted toward fatigue life
Fatigue cycling	2 ↔ 87.5 MPa	Advance CFRP fatigue life-fraction accumulation

**Table 9:** Comparison of cylinder-level structural fatigue life predicted using the P0 and P1 liner models.

Metric	P0 Elastoplastic	P1 Elastoplastic–Viscoelastic
Number of material parameters	4	10
Predicted structural fatigue life/cycles	17,617	21,136
Mean life of burst-tested cylinders/cycles	22,785	22,785
Absolute life deviation/cycles	5168	1649
Relative error with respect to the mean/%	22.68	7.24
Computational time per $\Delta N_{k}$/s	7062	8589
Computational-time change relative to P0	Baseline	+21.6%
Predicted critical region	Dome transition region near the equator	Dome transition region near the equator

**Table 10:** Changes in representative normalized load-carrying metrics before and after cycling.

Normalized Metric	Representative Ply	Initial N=0	Representative Late-Cycle State	Trend
$\sigma_{11}/X_{t}$	Ply 3, inner helical	0.357	0.205	Decrease
$\sigma_{11}/X_{t}$	Ply 16, outer hoop	0.308	0.423	Increase
$\sigma_{22}/Y_{t}$	Ply 3, inner helical	0.436	0.199	Decrease
$\sigma_{22}/Y_{t}$	Ply 16, outer hoop	0.759	0.300	Decrease
$\tau_{12}/S_{12}$	Ply 3, inner helical	0.047	0.450	Increase
$\tau_{12}/S_{12}$	Ply 17, outer helical	0.0056	0.194	Increase

**Table 11:** Experimental-numerical comparison of structural burst life.

Comparison Case	Cycle Count/Cycles	Deviation from Measured Mean Burst Life	Validation Purpose
V-1-#1 (burst)	23,213	—	Burst life and morphology
V-1-#2 (burst)	22,357	—	Burst life and morphology
V-1-#3 (valve leakage)	20,005	Not included in error calculation	Report competing termination mechanism and applicability
Mean burst life of #1 and #2	22,785	Reference	Structural fatigue-life reference
P0 prediction	17,617	22.68% lower	Liner-model comparison
P1 prediction	21,136	7.24% lower	Baseline for subsequent damage evolution

## Data Availability

The original contributions presented in this study are included in the article. Further inquiries can be directed to the corresponding authors.
